# MicroRNA-Mediated Regulation of Initial Host Responses in a Symbiotic Organ

**DOI:** 10.1128/mSystems.00081-21

**Published:** 2021-05-11

**Authors:** Silvia Moriano-Gutierrez, Edward G. Ruby, Margaret J. McFall-Ngai

**Affiliations:** a Pacific Biosciences Research Center, University of Hawaiʻi at Mānoa, Honolulu, Hawaiʻi, USA; University of Vienna

**Keywords:** noncoding RNA, symbiosis onset, *Euprymna scolopes*, *Vibrio fischeri*, development, immune response, developmental biology

## Abstract

One of the most important events in an animal’s life history is the initial colonization by its microbial symbionts, yet little is known about this event’s immediate impacts on the extent of host gene expression or the molecular mechanisms controlling it. MicroRNAs (miRNAs) are short, noncoding RNAs that bind to target mRNAs, rapidly shaping gene expression by posttranscriptional control of mRNA translation and decay. Here, we show that, in the experimentally tractable binary squid-vibrio symbiosis, colonization of the light organ induces extensive changes in the miRNA transcriptome. Examination of the squid genome revealed the presence of evolutionarily conserved genes encoding elements essential for the production and processing of miRNAs. At 24 h postcolonization, 215 host miRNAs were detected in the light organ, 26 of which were differentially expressed in response to the symbionts. A functional enrichment analysis of genes potentially targeted by downregulation of certain miRNAs at the initiation of symbiosis revealed two major gene ontology (GO) term categories, neurodevelopment and tissue remodeling. This symbiont-induced downregulation is predicted to promote these activities in host tissues and is consistent with the well-described tissue remodeling that occurs at the onset of the association. Conversely, predicted targets of upregulated miRNAs, including the production of mucus, are consistent with attenuation of immune responses by symbiosis. Taken together, our data provide evidence that, at the onset of symbiosis, host miRNAs in the light organ drive alterations in gene expression that (i) orchestrate the symbiont-induced development of host tissues, and (ii) facilitate the partnership by dampening the immune response.

**IMPORTANCE** Animals often acquire their microbiome from the environment at each generation, making the initial interaction of the partners a critical event in the establishment and development of a stable, healthy symbiosis. However, the molecular nature of these earliest interactions is generally difficult to study and poorly understood. We report that, during the initial 24 h of the squid-vibrio association, a differential expression of host miRNAs is triggered by the presence of the microbial partner. Predicted mRNA targets of these miRNAs were associated with regulatory networks that drive tissue remodeling and immune suppression, two major symbiosis-induced developmental outcomes in this and many other associations. These results implicate regulation by miRNAs as key to orchestrating the critical transcriptional responses that occur very early during the establishment of a symbiosis. Animals with more complex microbiota may have similar miRNA-driven responses as their association is initiated, supporting an evolutionary conservation of symbiosis-induced developmental mechanisms.

## Abstract (precis)

Animals often acquire their microbiome from the environment at each generation, making the initial interaction of the partners a critical event in the establishment and development of a stable, healthy symbiosis. However, the molecular nature of these earliest interactions is generally difficult to study and poorly understood.

## INTRODUCTION

Accommodation to a beneficial microbial symbiosis has been associated with pronounced changes in gene expression in host tissues (1–4), and recent studies have provided evidence that the activity of microRNAs (miRNAs) represents a major mechanism by which these processes are regulated (5, 6). This mode of regulation plays a crucial role in ensuring that proper gene expression patterns are established and maintained in each host cell type (7). miRNAs are small regulatory RNAs, constituents of the RNA-induced silencing complex (RISC), which regulate targeted genes via complementary binding to the 3′ untranslated region (3′ UTR) of their mRNA. Although target transcripts associated with such events are commonly downregulated by either inhibition of translation or mRNA degradation (7, 8), some studies have revealed that certain miRNAs are capable of activating gene expression directly or indirectly in a variety of cell types (9, 10). Regardless of their mechanism of action, miRNAs are known to be key regulators of biological processes such as early development, stress responses, apoptosis, and cell proliferation and differentiation (11, 12), as well as of host-microbe interactions (13, 14), which often are themselves inducers of these processes.

The miRNA biogenesis machinery, which often functions in response to symbiosis, is highly conserved among organisms (15). After miRNAs are transcribed from the genome, the primary transcripts are processed, first in the nucleus and then in the cytosol, by the RNase II enzymes Drosha and Dicer, respectively, ultimately generating a mature miRNA associated with the RISC complex (16). The RISC complex contains at its center an Argonaute/PIWI (AGO/PIWI) protein family member that is loaded with the mature miRNA sequence to form target recognition complexes (17, 18). Although miRNAs have been discovered in a wide variety of organisms, their characterization across the animal kingdom has generally focused on a restricted set of clades. The miRNA database (miRBase v22.1), for instance, encompasses 38,589 hairpin precursors (pre-miRNAs) in at least 271 different organisms (19). However, the collection of known miRNAs in the Lophotrochozoa, the subkingdom of the squid host studied here, is still relatively limited, with only 461 precursors identified in this group. Furthermore, within the phylum Mollusca, which includes squids, just 65 miRNA precursors are represented in the latest version of miRBase, and no miRNA high-throughput sequencing studies have been published to date for any members of the molluscan class Cephalopoda, which includes the octopods, squids, and their relatives.

**FIG 1:**
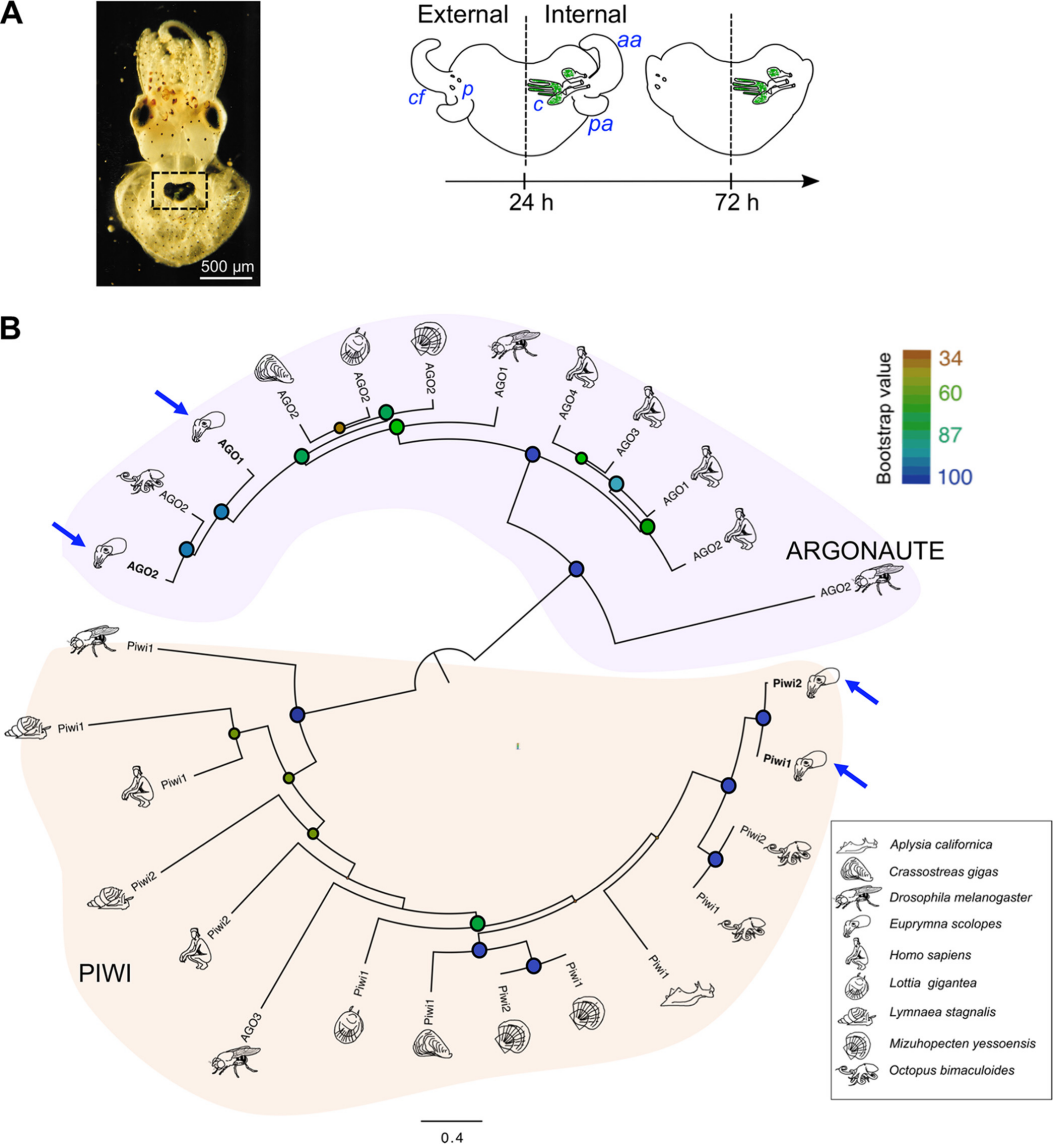
The molluscan Argonaute and PIWI gene repertoire. (A) The juvenile *E. scolopes*. (Left) Light organ (dotted box), seen through ventral mantle tissue. (Right) Early postembryonic development of the juvenile light organ. The light organ has 3 pores (*p*) that lead to the internal crypt spaces (*c*) where V. fischeri (green) is harbored. The surface tissues of the juvenile light organ include the anterior (*aa*) and posterior appendages (*pa*) that are covered by the ciliated field (*cf*). Both appendages regress during the first few days postcolonization. (B) Phylogenetic analysis of PIWI-like (Piwi) and Argonaute-like (AGO) proteins. Maximum-likelihood analysis bootstrap values of particular nodes are shown by blue to orange circles. Bar, amino acid substitution rates per site. Blue arrows indicate *E. scolopes* sequences.

To characterize miRNA-mediated gene regulation in response to symbiosis, we used as a model the highly specific mutualism between the Hawaiian bobtail squid, Euprymna scolopes, and the luminescent bacterium Vibrio fischeri. In this horizontally transmitted association, immediately following hatching, the nascent light organ is poised to interact with planktonic V. fischeri present in the surrounding seawater (20). Fields of cilia on the light organ surface (Fig. 1A) aid symbiont recruitment (21). Following 2 to 3 h of aggregation on the surface, the V. fischeri cells migrate to a set of surface pores, through which they enter host tissues. Over the subsequent hours, they migrate into the deep crypts of the light organ, where they proliferate and generate luminescence for the host’s behavior (22). Early in this crypt colonization, the symbionts trigger the irreversible loss of the ciliated field on the light organ surface through the induction of widespread apoptosis (23). In addition, the crypt epithelial cells that are in direct contact with the symbionts change shape and size (24), and the symbionts induce attenuation of certain immune responses of the host (25, 26). A recent study demonstrated that, associated with these phenotypic changes, V. fischeri affects the transcriptome of both the light organ itself (2, 3, 27), as well as tissues remote from the site of colonization, specifically the eye and the gill (3). Because this entire developmental program is triggered within the span of only 24 h, we have an opportunity to discover the series of molecular events that underpin its transcriptional regulation.

In this study, we compared miRNA expression profiles in uncolonized and colonized juvenile squid light organs at 24 h. Additionally, to provide more insight into the breadth of the E. scolopes miRNA repertoire, we compared the miRNAs present in the circulatory system of a symbiotic host to those found within the symbiotic light organ. Our data provide evidence that, upon colonization, the miRNA transcriptome in the light organ drives gene expression changes that orchestrate aspects of the developmental program of the symbiotic tissues and affect the host immune response to promote accommodation of the symbionts, two outcomes shared with other symbioses (28, 29).

## RESULTS

### Evolution and expression of the miRNA machinery of *E. scolopes*.

Using a phylogenetic framework, we analyzed the distribution of the guide RNA-protein repertoire of *E. scolopes* in the context of other mollusks, as well as fruit flies and humans as reference organisms. The Argonaute and PIWI protein sequence data were obtained from available annotations in NCBI of 6 different molluscan species, namely Aplysia californica, Crassostrea gigas, Lottia gigantea, Lymnaea stagnalis, Mizuhopecten yessoensis, and Octopus bimaculoides. The Argonaute and PIWI sequences of *E. scolopes* were found within existing transcriptional databases (3, 30). Unlike the octopus, which encodes only one Argonaute-like protein in its genome, *E. scolopes* has two putative orthologs that both cluster within the Argonaute (AGO) clade and are supported by high bootstrap values (Fig. 1B). PIWI family members, by contrast, are relatively conserved in cephalopods, where both octopus and *E. scolopes* have PIWI1- and PIWI2-like members (Fig. 1B). Furthermore, in agreement with previously reported gene trees, Drosophila melanogaster AGO3 shares a common ancestor with the PIWI-like clade members (31, 32). In summary, the data provide evidence that *E. scolopes* has evolutionarily conserved RNA-guided proteins within the Argonaute and PIWI gene families.

**FIG 2:**
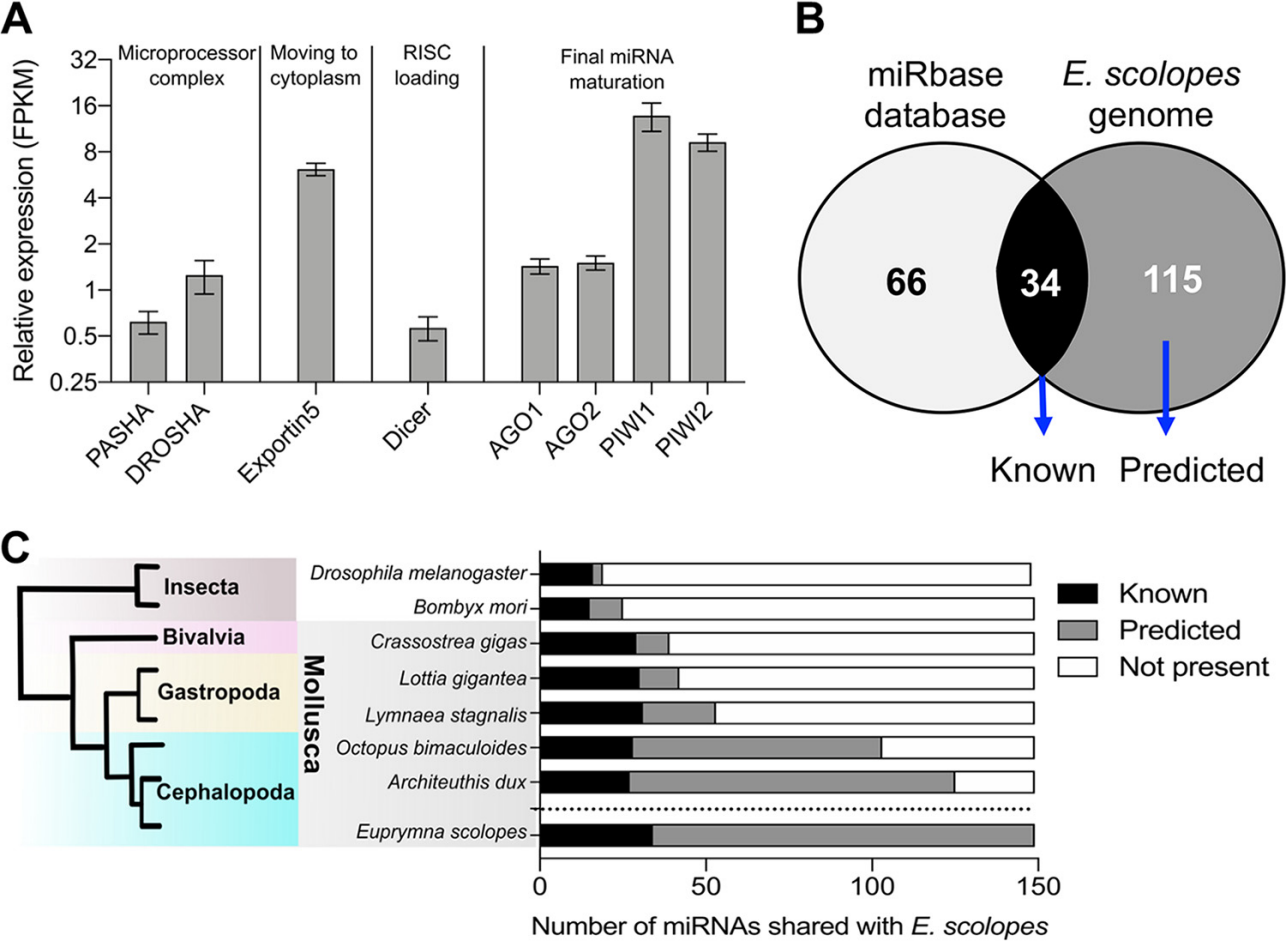
Expression of microRNA (miRNA) synthesis-associated proteins and the light organ miRNA database. (A) Light organ gene expression of proteins of the miRNA machinery 24 h posthatching, expressed as fragments per kilobase of transcript per million mapped reads (FPKM). Data are represented as the mean ± 1 standard deviation (SD) (*n* = 3). Expression data obtained from Moriano-Gutierrez et al. (3). (B) Venn diagram of miRNAs identified in the *E. scolopes* genome and miRbase database. (C) Number of miRNAs found in the *E. scolopes* genome that are shared across different organisms.

We also confirmed that additional proteins involved in the miRNA machinery are expressed in the light organ. Members of the microprocessor complex, namely Pasha (DGCR8 in vertebrates) and Drosha, the RISC-loading protein Dicer, and the miRNA guides AGO1 and AGO2, are expressed (Fig. 2A). Similarly, PIWI members and exportin 5, which are involved in the transport of the pre-miRNA to the cytoplasm for the final maturation steps, were expressed at relatively higher levels (Fig. 2A). Regardless of their expression level, transcripts of all major proteins of the miRNA machinery were present in the *E. scolopes* light organ, demonstrating that the squid has all the necessary components to deploy miRNA machinery for posttranscriptional regulation of gene expression in the symbiotic organ.

### Identification of known and predicted *E. scolopes* miRNAs in the squid light organ.

To study miRNA expression in the juvenile squid light organ, irrespective of symbiotic state, colonized (symbiotic [SYM]) or uncolonized (aposymbiotic [APO]) organs were collected for small RNA sequencing at 24 h postinoculation, the approximate time that the symbiosis first becomes fully functional. After removing contaminating V. fischeri sequences and well-characterized host noncoding RNAs (rRNAs, tRNAs, and snoRNAs), all remaining reads ranging from 14 to 36 bp were collected for alignment with both the *E. scolopes* genome (30) and the latest miRBase database (v22.1) using miRDeep2 software (33). An analysis of the squid genome and the miRBase database identified a total of 215 miRNAs among the combined SYM and APO juvenile light organ samples (see Table S1 in the supplemental material). Of these identified miRNAs, 66 were found only in the miRBase database and, although they had been isolated from the squid host, could not be identified within the *E. scolopes* genome (see Data Set S1 in the supplemental material). Reports of extracted miRNAs that cannot be mapped to the genome of an animal (or its symbiont) have identified them in the animal’s diet (34); however, because the juvenile squid used here had not fed in the first 24 h posthatching, the unidentified miRNAs are more likely to be encoded in the squid genome but unrecognized.

10.1128/mSystems.00081-21.4 TABLE S1 Numbers of miRNAs detected in squid light organ and hemolymph. Download Table S1, DOCX file, 0.03 MB. Copyright © 2021 Moriano-Gutierrez et al. 2021 Moriano-Gutierrez et al. https://creativecommons.org/licenses/by/4.0/ This content is distributed under the terms of the Creative Commons Attribution 4.0 International license.

10.1128/mSystems.00081-21.10 DATA SET S1 (Sheet 1) List of *E. scolopes* light organ miRNAs. (Sheet 2) List of “known” *E. scolopes* light organ miRNAs found only in miRBase. (Sheet 3) List of miRNAs found in squid hemolymph. (Sheet 4) List of “known” *E. scolopes* hemolymph miRNAs found only in miRBase. Download Data Set S1, XLSX file, 0.05 MB. Copyright © 2021 Moriano-Gutierrez et al. 2021 Moriano-Gutierrez et al. https://creativecommons.org/licenses/by/4.0/ This content is distributed under the terms of the Creative Commons Attribution 4.0 International license.

Among the 149 miRNAs that could be localized in the genome, 34 were found within the miRBase database and were designated “known” miRNAs, while the remaining 115 miRNAs were not found within miRBase and were therefore considered to be “predicted” (Fig. 2B and Data Set S1). To characterize the miRNA light organ database more fully, we compared the miRNAs found in the squid genome to those of five other mollusk genomes, C. gigas, L. gigantea, L. stagnalis, O. bimaculoides, and Architeuthis dux, as well as to those of two insects, D. melanogaster and Bombyx mori, as outgroups. As expected, the number of matching predicted miRNA sequences increased with phylogenetic proximity (Fig. 2C), with only 24 of the 115 (∼21%) predicted miRNAs specific to the *E. scolopes* lineage. Similarly, most of the 34 known miRNAs were present among all the other molluscan species examined, but only 50% of these miRNAs were also identified in the two insects.

### Comparison of the light organ miRNAs with those present in the circulation.

Given the relatively low number of conserved (i.e., “known”) miRNAs expressed in the light organ (Fig. 2B), we asked whether this scarcity was a characteristic specific to this organ or, instead, was a general feature of *E. scolopes* tissues. Thus, to sample a broader miRNA population that was likely to provide a better reflection of the miRNA repertoire of the entire animal (35), we performed a transcriptome sequencing (RNA-seq) analysis of the miRNAs circulating in the squid hemolymph.

While a sufficient volume of hemolymph could not be obtained from juvenile animals, we were able to draw hemolymph samples from two adult wild-caught squid and process them for miRNA analysis. A total of 268 predicted miRNAs, but only 18 known miRNAs, were identified in the circulating miRNA population (Table S1 and Data Set S1). Thus, although only 23% of the light organ miRNAs encoded in the genome were known, an even smaller percentage (6%) of the hemolymph miRNAs were classified as known (Table S1), suggesting that the high percentage of predicted (i.e., not found in miRBase) miRNAs expressed by *E. scolopes* tissues is not a characteristic unique to the light organ.

To determine whether members of the population of light organ miRNAs could be detected within the hemolymph as well, we compared the miRNAs isolated from these two locations. All 18 known (i.e., present in miRBase) miRNAs identified in circulation were also expressed in light organ tissues. However, only 46 of the 268 predicted miRNAs in hemolymph were found both in the light organ and in circulation, suggesting that the circulation was sampling a large number of novel tissue-specific miRNAs from the other organs it served. Support for this hypothesis came from the finding that 69 and 222 of the predicted miRNAs were specific to the light organ and hemolymph, respectively (Table S1; see also Fig. S1A in the supplemental material). Principal-component analysis (PCA) of the miRNA profiles revealed that sample origin was the primary factor affecting global miRNA expression; i.e., principal component 1 (PC1; 68% of the overall variance) separated light organ from hemolymph samples, while PC2 (only 11% of the overall variance) separated symbiotic from aposymbiotic samples (Fig. S1B). When comparing expression values of individual miRNAs, samples clustered strongly by symbiotic state (Fig. S1C); significantly, the number of differentially expressed miRNAs shared between hemolymph and aposymbiotic light organ samples (*n* = 55) was nearly double that between hemolymph and colonized light organs (*n* = 29) (see Table S2 in the supplemental material), again indicating that the presence of the symbionts is the main variable driving the populations of both tissue-specific (i.e., light organ) and circulating miRNAs.

10.1128/mSystems.00081-21.1 FIG S1 Profiling of the circulating microRNAs (miRNAs). (A) Comparison between predicted miRNAs identified in adult squid hemolymph and predicted miRNAs identified in juvenile light organs. (B) Principal component analysis (PCA) of expression profile of light organ miRNAs and hemolymph miRNAs; HEM, hemolymph; APO, aposymbiotic light organ; SYM, symbiotic light organ. (C) Heatmap of expression profile of miRNAs differentially expressed between light organ and hemolymph (false-discovery rate (FDR, <0.05; fold change, >2). The miRNA identifiers (IDs) listed can be found in Table S2. Download FIG S1, TIF file, 0.4 MB. Copyright © 2021 Moriano-Gutierrez et al. 2021 Moriano-Gutierrez et al. https://creativecommons.org/licenses/by/4.0/ This content is distributed under the terms of the Creative Commons Attribution 4.0 International license.

10.1128/mSystems.00081-21.5 TABLE S2 List of differentially expressed miRNA between the light organ (aposymbiotic [APO], symbiotic [SYM]) and the hemolymph (HEM). Download Table S2, DOCX file, 0.04 MB. Copyright © 2021 Moriano-Gutierrez et al. 2021 Moriano-Gutierrez et al. https://creativecommons.org/licenses/by/4.0/ This content is distributed under the terms of the Creative Commons Attribution 4.0 International license.

### The differential expression profile of miRNAs in response to light organ symbiosis.

**FIG 3:**
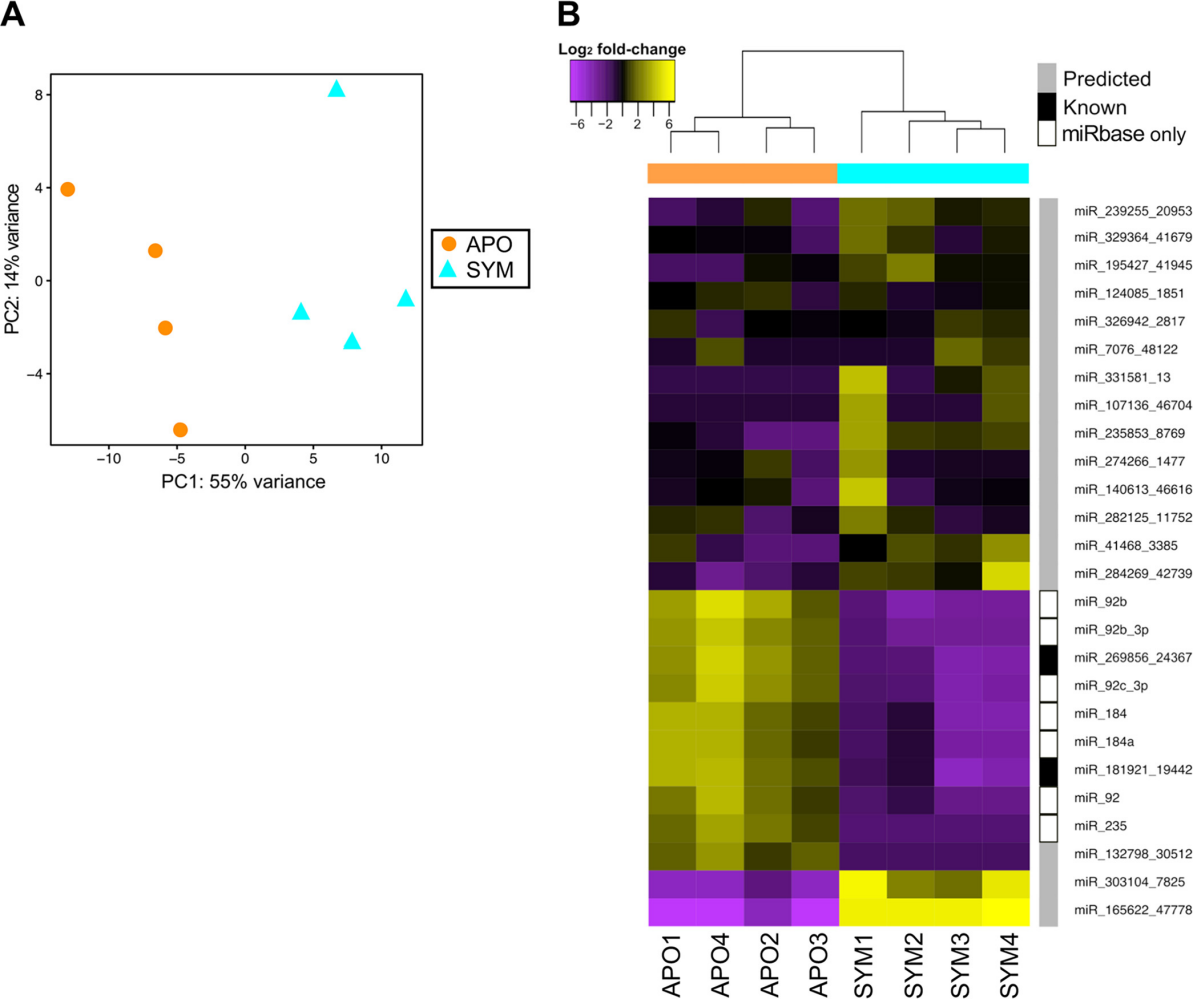
Effect of symbiosis on light organ miRNA profile. (A) Principal-component analysis (PCA) of miRNA gene expression. A PCA scatterplot shows the variance of the four biological replicates of colonized (symbiotic [SYM]) or uncolonized (aposymbiotic [APO]) light organs. The percentage on each axis indicates the degree of variation explained by the principal components. (B) Heatmap of expression values of the light organ miRNAs that are differentially expressed (false-discovery rate [FDR], <0.05; fold change, >2) in response to symbiosis (*n* = 4). M19, miR_326942_2817. Each sample corresponds to 20 pooled light organs (see Materials and Methods). The bar to the left of the names of the miRNAs indicates the status of the miRNA as follows: gray box, predicted in the genome; black box, known; or white box, only present in miRbase but not detected within the genome. See Fig. 2B.

To determine the effect of symbiosis onset on the miRNA population, the expression of miRNAs in SYM light organs at 24 h was compared more comprehensively to that in APO light organs. A PCA using the light organ-derived data only revealed that colonization state was the primary factor affecting global miRNA expression in squid light organs; i.e., PC1 (55% of the overall variance) separated APO animals from SYM animals (Fig. 3A). A total of 26 light organ miRNAs were differentially expressed; specifically, 16 miRNAs were upregulated and 10 miRNAs were downregulated with symbiosis (Fig. 3B and Table S3 in the supplemental material). Interestingly, only two known (i.e., present in miRBase) miRNAs change their expression levels with symbiosis, and both are downregulated; in contrast, 16 of 17 predicted (i.e., not present in miRBase) miRNAs were upregulated with symbiosis, suggesting that, in the light organ, the symbiotic state requires a response dominated by miRNAs unique to the *E. scolopes* clade. In addition, because the typical response to miRNA is the downregulation of transcription, the predominant effect of this regulatory mechanism in symbiosis appears to be to reduce gene expression in the light organ. Two of these host-specific, symbiosis-induced miRNAs were also present in the hemolymph.

10.1128/mSystems.00081-21.6 TABLE S3 List of light organ miRNAs that are differentially expressed with symbiosis. Download Table S3, DOCX file, 0.03 MB. Copyright © 2021 Moriano-Gutierrez et al. 2021 Moriano-Gutierrez et al. https://creativecommons.org/licenses/by/4.0/ This content is distributed under the terms of the Creative Commons Attribution 4.0 International license.

### Target prediction for light organ-regulated miRNAs.

**FIG 4:**
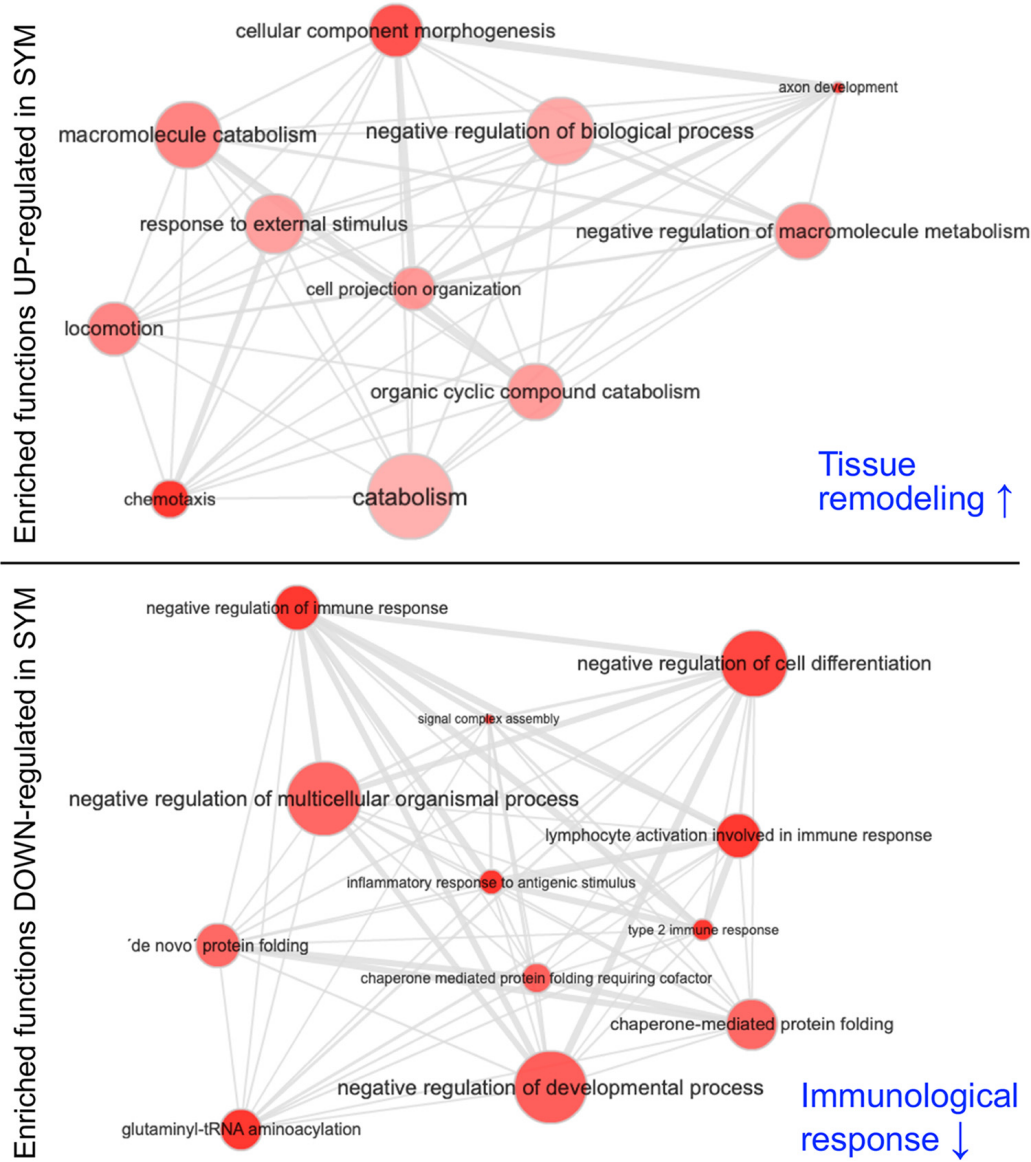
Functional enrichment analysis of the predicted miRNA target genes. Gene ontology (GO) enrichment of predicted mRNA targets of differentially expressed miRNAs present in the *E. scolopes* genome. Darker red color indicates higher statistical significance of GO terms; larger bubble size indicates higher frequency of the specific term in the Gene Ontology Annotation (GOA) Database. Gray lines link highly similar GO terms, where the width of the line indicates the degree of similarity. Predicted outcomes driven by symbiosis are shown in blue.

To identify the possible function(s) underlying miRNA regulation in response to light organ symbiosis, candidate mRNA targets were predicted with miRanda software using sequence complementarity. The 10 miRNAs that are upregulated in APO animals (i.e., are downregulated when the animal becomes SYM) had 108 predicted mRNA targets, 64% of which are targets of the two known regulated miRNAs (Fig. 3), miRNA_269856_24367 and miRNA_181921_19442, with 28 and 41 potential targets, respectively. Similarly, the 16 miRNAs upregulated in SYM animals had 188 predicted mRNA targets (see Table S4 in the supplemental material. For each group of targeted mRNAs, a functional enrichment analysis using annotated gene ontology (GO) terms was performed (Fig. 4 and Table S5 in the supplemental material). Targets of those miRNAs that are upregulated in symbiotic animals were enriched in immune responses, with associated frequent keywords such as “immunological,” “immunogenic,” or “stimulus” (Fig. 4 and Fig. S2 in the supplemental material). As miRNAs typically downregulate gene expression, these data indicate that light organ symbiosis turns down host tissue immune responses. In contrast, targets of miRNAs that are downregulated in symbiotic animals are enriched in neurodevelopmental functions, with associated frequent keywords such as “chemotaxis,” “migration,” “pathfinding,” or “cytoskeleton directed,” which in turn indicates that the establishment of symbiosis leads to an upregulation of tissue remodeling activities (Fig. 4 and Fig. S2).

10.1128/mSystems.00081-21.2 FIG S2 Functional enrichment analysis of predicted miRNA target genes. Gene ontology (GO) enrichment of predicted mRNA targets of differentially expressed miRNAs present in the *E. scolopes* genome. Tag cloud displaying overrepresented words in the descriptions of the GO terms, with larger and darker letters signifying stronger overrepresentation. Download FIG S2, TIF file, 0.3 MB. Copyright © 2021 Moriano-Gutierrez et al. 2021 Moriano-Gutierrez et al. https://creativecommons.org/licenses/by/4.0/ This content is distributed under the terms of the Creative Commons Attribution 4.0 International license.

10.1128/mSystems.00081-21.7 TABLE S4 Predicted mRNA targets of miRNAs differentially upregulated in either the aposymbiotic (APO) or symbiotic (SYM) state. Download Table S4, DOCX file, 0.1 MB. Copyright © 2021 Moriano-Gutierrez et al. 2021 Moriano-Gutierrez et al. https://creativecommons.org/licenses/by/4.0/ This content is distributed under the terms of the Creative Commons Attribution 4.0 International license.

10.1128/mSystems.00081-21.8 TABLE S5 List of significantly enriched functions in predicted mRNA targets of regulated miRNAs. Download Table S5, DOCX file, 0.1 MB. Copyright © 2021 Moriano-Gutierrez et al. 2021 Moriano-Gutierrez et al. https://creativecommons.org/licenses/by/4.0/ This content is distributed under the terms of the Creative Commons Attribution 4.0 International license.

### Validation of RNA-seq data by qRT-PCR.

**FIG 5:**
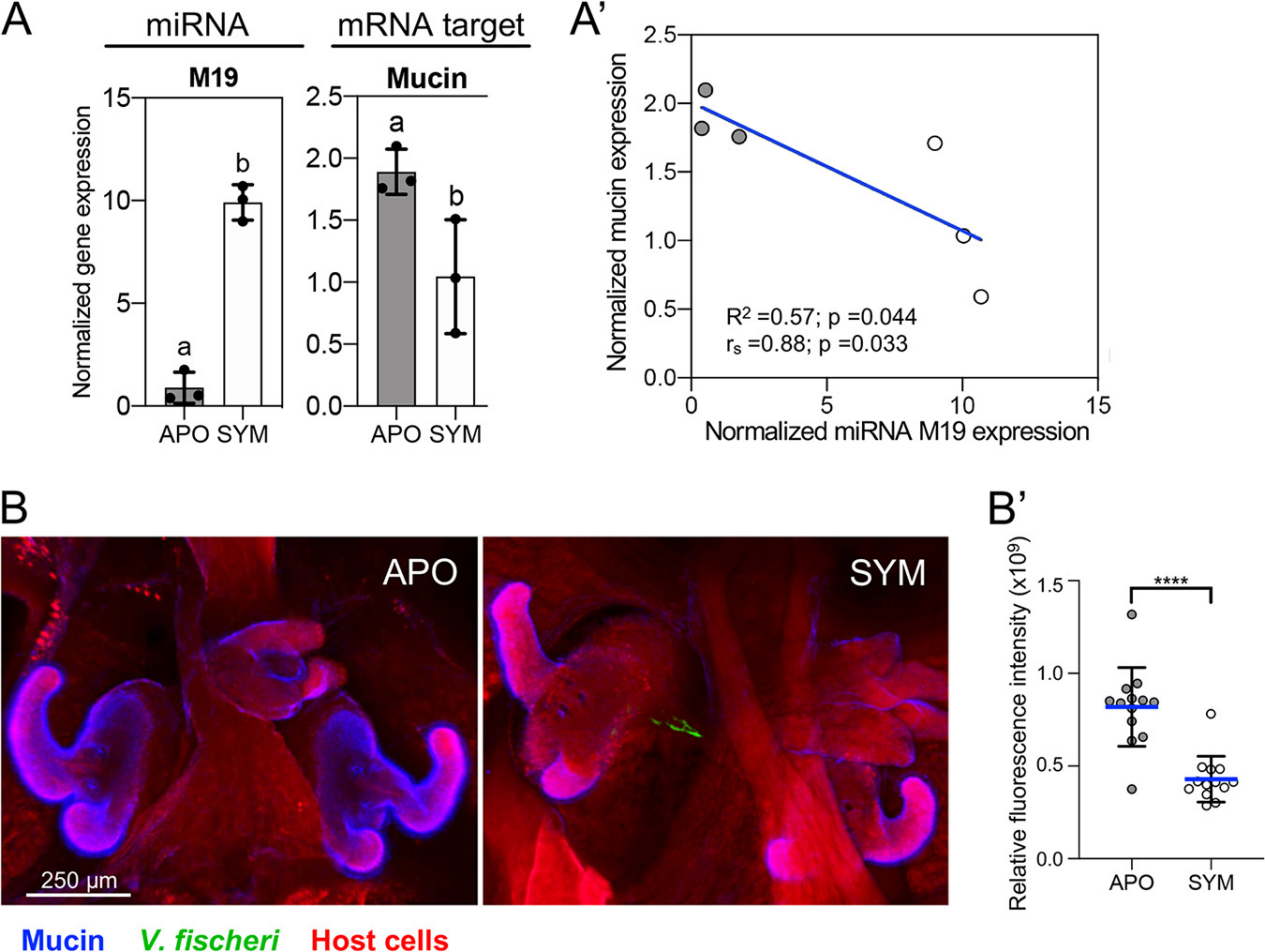
Regulation of mucin secretion by an miRNA. (A) Expression values of miRNA M19 and its potential mucin-encoding mRNA target by reverse transcription-quantitative PCR (qRT-PCR). Expression was normalized to U6 RNA for miRNA expression values (left) and to ribosomal protein S19 for the mucin mRNA targets (right). M19, miR_326942_2817. Data are represented as the mean ± 1 SD. Significant differences are indicated by an unpaired *t* test (*n* = 3). Each sample corresponds to 20 pooled light organs. (A′) The correlation of expression between miRNA and mRNA of symbiotic (SYM; white) and aposymbiotic (APO; gray) was analyzed by both Spearman correlation (*r_s_*) and linear regression (*R*^2^). *P* value (p) of <0.05 were considered significant. (B) Z-stack representative confocal image of an APO or SYM light organ, indicating higher secreted mucin staining (blue; wheat germ agglutinin, Alexa 633) in APO crypts, and green fluorescent protein (GFP)-labeled V. fischeri (green) only in SYM crypts; host cells (red; CellTracker). (B′) Quantification of mucin signal using the relative fluorescence intensity of light organ Z stacks (*n* = 14); error bar = SD; *P* < 0.0001 (****; Mann-Whitney test).

The expression changes of five differentially expressed miRNAs and their potential mRNA targets, selected from APO or SYM light organs, were measured by reverse transcription-quantitative PCR (qRT-PCR). These miRNAs were selected for validation based on both their relatively high expression level (over 6 counts per million), and the annotated functions of their respective mRNA targets. Briefly, using specific primers (see Table S6 in the supplemental material), three out of the five of these miRNAs were expressed at significantly different levels, depending on colonization state, compared to the RNA-seq expression data (Fig. 5A; see also Fig. S3 and Table S3 in the supplemental material); the other two, M9 and M25, while not significantly different, showed the same trend (Fig. S3 and Table S3). As miRNAs typically downregulate the expression of their targets, we measured the expression levels of the potential targets of these miRNAs to identify those with negative correlation between the transcription of miRNAs and their predicted target transcripts. We confirmed with confidence the negative correlation in expression of three pairs of miRNAs and their predicted targets (Fig. 5A and Fig. S3). Although the confirmed negative correlations are supportive, this finding is still only an indication of the potential interaction between miRNAs and transcript targets, which may explain why correlations are sometimes not proportional, as in the cases of M24 and M25 (Fig. S3).

10.1128/mSystems.00081-21.3 FIG S3 Expression values of selected regulated miRNAs and their potential mRNA targets by reverse transcription-quantitative PCR (qRT-PCR). Expression was normalized to U6 RNA for miRNA expression values (left) and to ribosomal protein S19 for mRNA targets (middle). Sialin and NaCa exchanger are involved in membrane efflux, chorion peroxidase functions in egg development, and zinc 420 is a transcription factor. Data are represented as the mean ± 1 standard deviation (SD). Significant differences are indicated after an unpaired *t* test (*n* = 3). Each sample corresponds to 20 pooled light organs. Expression correlation between miRNA and mRNA was analyzed by both Spearman correlation (*r_s_*) and linear regression (*R*^2^). *P* < 0.05 was considered significant (right, bold values). M9, miR_132798_30512; M11, miR_140613_46616; M25, miR_107136_46704; M2, miR_7076_48122. Download FIG S3, TIF file, 0.3 MB. Copyright © 2021 Moriano-Gutierrez et al. 2021 Moriano-Gutierrez et al. https://creativecommons.org/licenses/by/4.0/ This content is distributed under the terms of the Creative Commons Attribution 4.0 International license.

10.1128/mSystems.00081-21.9 TABLE S6 List of oligonucleotides. Download Table S6, DOCX file, 0.03 MB. Copyright © 2021 Moriano-Gutierrez et al. 2021 Moriano-Gutierrez et al. https://creativecommons.org/licenses/by/4.0/ This content is distributed under the terms of the Creative Commons Attribution 4.0 International license.

### Regulation of mucin secretion by miRNAs.

Mucus, composed of the principal protein, mucin, is a host secretion that provides a matrix within which V. fischeri cells from the seawater aggregate and form a biofilm during precolonization events (36). We observed that miRNA M19 is upregulated with colonization of the light organ, while the potential target that encodes mucin production is downregulated (Fig. 5A). This finding was supported by confocal microscopy visualization of the extent of mucus secretion by the host (Fig. 5B), i.e., the amount of mucus accumulating on the ciliary surfaces of the light organ was significantly reduced when the light organ is colonized (Fig. 5B′), suggesting that this response to colonization is mediated in part by miRNA.

## DISCUSSION

In this study, we first confirmed that all critical elements of the miRNA-production machinery are present and expressed in *E. scolopes* tissues. We identified both known and novel miRNAs associated with the onset of symbiosis in the juvenile host light organ and compared these miRNA populations to those of host hemolymph to get a broader idea of the miRNA repertoire of *E. scolopes*. We linked colonization-induced changes in the miRNA population to expression changes in the transcriptional responses that are among those previously observed in the symbiotic light organs (2, 3, 27). Taken together, these data provide evidence that miRNAs in the squid-vibrio symbiosis play a central role in controlling two major host phenotypes during early symbiosis, pronounced tissue morphogenesis and immune suppression.

### miRNAs identified in *E. scolopes* and their relationship to those of other animals.

The number of miRNAs described in animal genomes ranges widely, from ∼2,000 in most vertebrates (37) to only a few hundred in some invertebrates (12, 38). The presence in other animals of orthologs of E. scolopes miRNAs reflects their conservation across diverse taxa; these orthologs are found in miRBase and are described here as “known” miRNAs. Nearly 88% of the known miRNAs found within the *E. scolopes* genome show sequence conservation within the Mollusca, and 50% appear across even larger evolutionary distances (Fig. 2C). In contrast, the subset of ∼50 predicted miRNAs that is uniquely shared among the three cephalopods O. bimaculoides, A. dux, and *E. scolopes* likely comprises as yet undescribed miRNAs specific to this class of mollusks.

Approximately 35% and 46% of the total miRNAs identified in the *E. scolopes* light organ or hemolymph, respectively, are known (see Table S1 in the supplemental material), yet only 23% of those detected in the light organ were found within the squid genome. These percentages of known miRNAs are similar to those reported in two other mollusk genomes (39, 40), although the relative proportion of such conserved miRNAs varies greatly (∼7% to ∼50%) among high-throughput sequencing studies of mollusks (39–43). Such variation might be attributed to the different levels of completeness for each mollusk genome, sequencing batch biases, or the miRBase database used for the analyses, each of which will influence the efficiency with which new miRNA families are discovered. Similarly, we recognize that, as in all such studies, the number of miRNAs identified in the *E. scolopes* genome here may be over- or underestimated, i.e., identification of potentially orthologous miRNAs extrapolated from other mollusk genomes by sequence conservation and minimum free energy of hairpin structures does not necessarily imply that these apparent orthologs retain a similar function in *E. scolopes*. Alternatively, authentic orthologs present in distant mollusk species may have accumulated too many substitutions to be identified in the *E. scolopes* genome by sequence complementarity.

**FIG 6:**
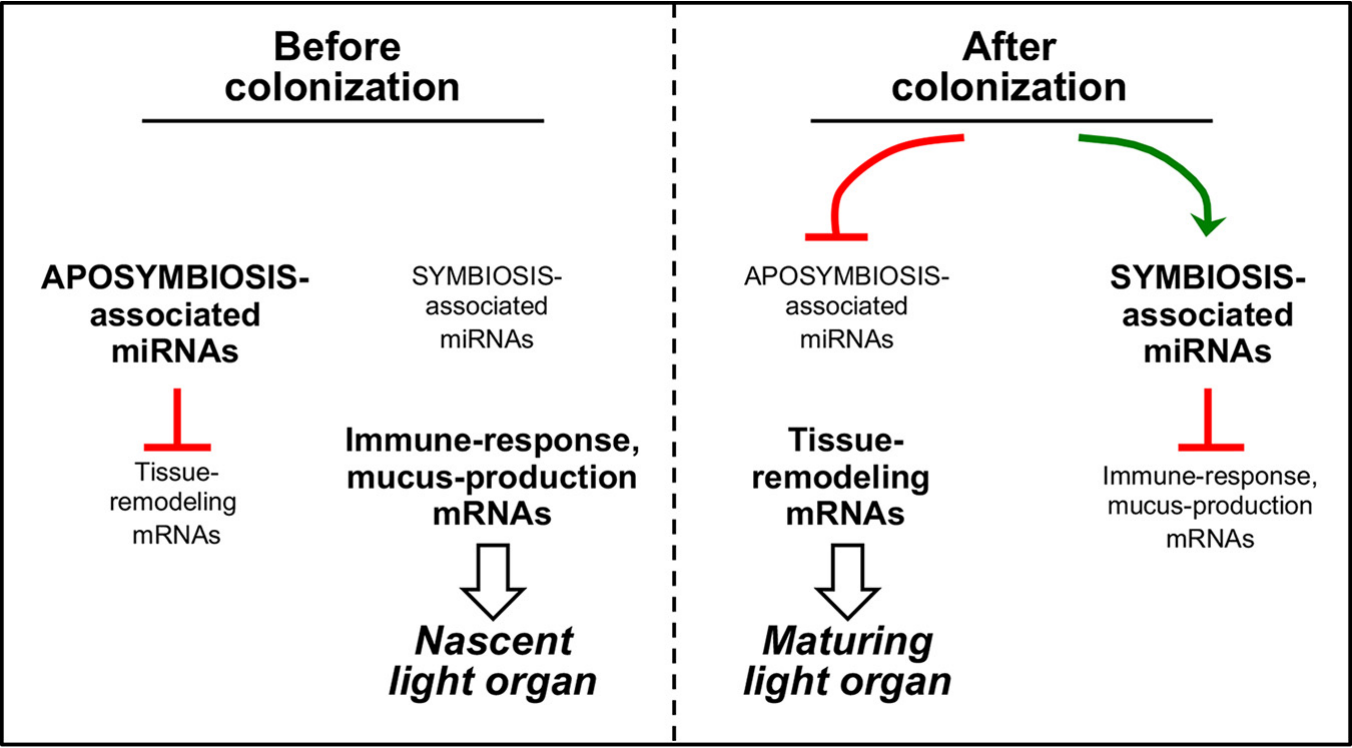
Summary diagram illustrating the proposed control of symbiotic colonization and development by host microRNAs (miRNAs). The tissues of the aposymbiotic light organ in a newly hatched juvenile squid (i) maintain a capacity to immunologically resist infection by nonspecific bacteria through the expression of particular mRNAs and (ii) use a set of miRNAs to restrict the expression of genes involved in initiating the maturation of a functional organ. Upon colonization by V. fischeri cells, these host miRNAs are downregulated, releasing the light organ to begin to develop the association; conversely, a new set of miRNAs are induced that suppress the organ’s immunological defenses, thereby facilitating the establishment of symbiosis.

In *E. scolopes*, 21% of the “predicted” miRNAs are lineage specific (Fig. 2C). While many of these miRNAs may have arisen through neutral processes, some might reflect specific evolutionary events leading to the acquisition of a symbiotic light organ by sepiolid squids (44); selection for such a complex organ is likely to have required the development of additional regulatory mechanisms, like novel miRNAs, to control the functions of the symbiosis. Consistent with this hypothesis are the findings that (i) all of the miRNAs whose expression is increased in colonized light organs are novel/predicted, and (ii) the majority of these are apparently specific to *E. scolopes;* i.e., only 23% and 31% of them are also present in the non-light-organ-bearing species O. bimaculoides and *A. dux*, respectively (Fig. 2C). Furthermore, these miRNAs are differentially expressed in the light organ and hemolymph of the symbiotic (SYM) host compared to the aposymbiotic (APO) host, indicating that they belong to a symbiosis-specific response (Fig. 5 and 6). Taken together, the data provide evidence that the symbiosis has driven the expansion of components of the host’s miRNA repertoire.

In a recent landscape study of several organs in mammals, the majority of tissue-specific miRNAs were novel/predicted (35, 45). While only the light organ was examined here, a significant percentage (41%) of its total expressed miRNAs were also predicted (Table S1). Perhaps more striking was the high number (268) and percentage (65%) of predicted miRNAs among the total sequences present in the squid’s circulating hemolymph (Table S1), into which miRNAs expressed in various tissues throughout the body can find their way (46). In mammals, miRNAs in the blood influence gene expression and activity in distinct tissues across the body (47–49). Similarly, miRNAs found in the squid hemolymph may mediate the series of symbiosis-induced changes in gene expression reported to occur in organs situated remotely to the light organ (3). Given the evidence of RNA stability in different body fluids (47, 48, 50, 51) and its rapid propagation throughout the body within extracellular vesicles (52), we anticipate that circulating miRNAs will be found to play a regulatory role in a variety of host-microbe associations.

### The miRNA signature of symbiosis onset.

The data presented here provide a window into understanding the role of miRNAs in early gene regulation of symbioses in general. For instance, in the last decade, investigation of a variety of plants and animals has highlighted the importance of miRNAs in posttranscriptional regulation; notably, in some cases, miRNAs have been shown to be essential for the symbiotic state (53–59). However, to date, only in the well-studied legume-rhizobia root nodule symbioses has the role of gene regulation by miRNAs been analyzed in detail throughout the ∼20-day trajectory from initiation to a functioning symbiotic organ (60, 61).

A critical difference in the analyses of miRNAs in animal associations is that the time course of symbiosis onset is typically abbreviated compared to that of interactions of plants in the root nodule or with mycorrhizae (62); i.e., rather than taking days, animals can establish a functional symbiotic association within minutes to hours of encountering their symbionts. For example, analyses of the initial microbiomes of skin, oral mucosa, and nasopharynx of human neonates within 5 min of birth revealed the nature of the microbial founder populations, as did analyses of the gut microbiota in the meconium at 24 h (63). Having described the composition of these initial communities, an obvious question is how do the transcriptomes of host tissues respond to these early events, and what regulatory mechanisms (e.g., miRNAs) drive these responses? Several miRNA studies have shown differences between germfree and colonized mice; for example, when 8-week-old germfree mice were exposed to microbiota for several days, the host tissues reprogramed their transcription through miRNAs (5, 64). Similarly, miRNAs present in breast milk rapidly shape the microbiome of the gut by playing a key role in immunomodulation (65), a common event in the establishment of beneficial symbioses (e.g., references 66–68).

While investigating the regulation of early transcriptional events in the squid-vibrio association, we found that, except for one, all the miRNAs that were downregulated in colonized animals are in the “known” and “miRbase only” categories (Fig. 3B); i.e., they are widely conserved among other organisms (Fig. 2C). Because the expression of the mRNA targets of these miRNAs would be predicted to be increased in symbiotic light organs, we hypothesize that the upregulation of these genes in response to colonization is an evolutionarily conserved phenomenon. In support of this notion, orthologs of these known miRNAs, including members of the miR-92 and miR-184 families, have been implicated in controlling gene expression in diverse host-microbe associations (14, 54, 69). However, the direction of their regulation varies depending on the context, particularly between beneficial and pathogenic associations. Specifically, miR-92 family members are generally upregulated in response to pathogenesis (69–73), while, similarly to their orthologs in the light organ, they are downregulated in response to beneficial symbiont metabolites (74), Wolbachia or Buchnera colonization (13, 75), or Toll-like receptor (TLR) activation (76). Thus, members of the miR-92 family of miRNAs may play key roles in regulating host responses to both beneficial and pathogenic interactions.

### Defining putative targets of symbiosis-induced miRNAs.

To identify the possible functions of the differentially expressed miRNAs, putative target genes were predicted using the 3′ UTR regions in the *E. scolopes* transcriptome (30). These presumed targets were then subjected to a GO enrichment analysis to classify their expected functions. The results indicated that targets of miRNAs upregulated in symbiosis are enriched in genes that function to attenuate immune responses, with associated frequent keywords such as “immunological,” “immunogenic,” or “stimulus” (Fig. 4). As miRNAs typically downregulate the expression of their target genes, this result suggests that, upon colonization, host cells within the light organ downregulate their immune response. Consistent with this finding, V. fischeri colonization triggers, within the light organ, a dramatic decrease in both nitric oxide (NO) and NO synthase (25), as well as in laccase (67) and halide peroxidase (26), all antimicrobial immune effectors. The underlying mechanism(s) of such responses had previously remained unexplored, yet, based on our findings, posttranscriptional regulation by miRNAs might be key to achieving symbiotic homeostasis. In support of this notion, in beneficial plant-microbe interactions, the majority of pathways targeted by miRNAs during the establishment of symbiosis are related to turning off the defense pathways of the host (57). We confirmed a negative correlation between the expression of miRNA M19 and its predicted target, the gene encoding mucin synthesis (Fig. 5A). Mucin is a component of mucus, a host-derived secretion that is well known for helping clear pathogens (77) and build symbiosis-enhancing structures (28), and in the light organ symbiosis, aids the selection of V. fischeri cells during initiation (36). Upon successful colonization, mucus secretion is reduced on the surface of the ciliary field of the light organ (Fig. 5B and C), in agreement with the miRNA expression data.

In contrast, targets of miRNAs downregulated by symbiosis are enriched in genes affiliated with tissue remodeling and neurodevelopment, with associated frequent keywords such as “cytoskeleton directed,” “chemotaxis,” or “migration.” As colonization of the light organ induces morphological changes in the organ tissues (78), it is not unexpected that symbiosis would enhance expression of mRNAs whose products are involved in tissue differentiation (28) (Fig. 6). While symbiosis-induced tissue remodeling and changes in cell morphology have been well established among various organisms (79–81), the underlying molecular mechanisms of such host responses have remained elusive. However, there are some indications that miRNAs are involved. For instance, in germfree mice, symbiotic colonization of the gut tract increases the renewal rate of crypt intestinal cells and is reflected by changes in host epithelial gene expression (82). Recently, miRNAs were implicated in the regulation of epithelial cell physiology, indicating that miRNAs are essential modulators of intestinal homeostasis with important roles in cell proliferation and differentiation (83, 84). Furthermore, downregulation of one miRNA (miR-375) by symbiosis significantly increases the proliferative capacity of intestinal epithelial cells, providing a potential mechanism by which the microbiota induces cell proliferation *in vivo* (85).

### Conclusions.

In future studies of the squid-vibrio symbiosis, the goal will be to elucidate the regulatory networks of miRNA-driven changes in gene expression that sustain a symbiosis over the various developmental milestones of host ontogeny. Principles derived from interpreting the conversation between a host and its monospecific association have provided useful guideposts for understanding the development of the more complex consortial communities present in most animals (78). As such data become available for these other hosts, including humans, comparisons with the patterns of miRNA gene regulation in the squid-vibrio system will similarly provide a window into the extent to which these patterns are conserved across the animal kingdom.

## MATERIALS AND METHODS

### Squid light organ colonization assays.

The breeding colony of Hawaiian bobtail squid (Euprymna scolopes) was collected from Maunalua Bay, Oahu, Hawaii, and maintained in flowthrough seawater tanks on a natural 12-h:12-h light-dark cycle in the Kewalo Marine Laboratory. Within 2 h of hatching, juvenile squid were either exposed overnight to V. fischeri cells (i.e., wild-type strain ES114 [86]) at a concentration of 3,000 to 6,000 CFU/ml overnight (SYM), or kept aposymbiotic (APO) in filter-sterilized ocean water (FSOW). Bacterial cells were cultured overnight in Luria-Bertani salt medium (LBS) (87), subcultured into seawater tryptone medium (SWT) (86), and grown to the mid-log phase at 28°C with shaking at 220 rpm. Colonization of the host was determined by monitoring animal luminescence with a TD20/20 luminometer (Turner Designs, Sunnyvale, CA). After 24 h of colonization, squid were anesthetized in seawater containing 2% ethanol and stored at −80°C in RNAlater (Sigma-Aldrich) until further processing.

### Isolation and sequencing of RNA from hemolymph.

Adult wild-caught squid were anesthetized with 2% ethanol in seawater prior to hemolymph extraction from the cephalic artery. Each squid was sampled only once at either 4 p.m. or 2 a.m., and between 200 and 300 μl of hemolymph was recovered. Circulating hemocytes were removed by centrifuging the samples at 5,000 rpm for 10 min at 4°C to pellet the cells. Pooled cell-free hemolymph from two adults was used for RNA purification at each time point. Total extracted RNA was purified using the mirVana Paris kit (Invitrogen), which was followed by treatment with DNase I (Thermo Fisher Scientific). The RNA concentration was determined with a Qubit RNA broad-range (BR) assay kit (Invitrogen). The libraries of the small RNA components were constructed using the TruSeq small RNA sample preparation kit according to the manufacturer’s instructions. The quality of the RNA libraries was assessed with the Agilent 2100 Bioanalyzer system. Single-end 50-cycle sequencing was performed using an Illumina HiSeq 2500 platform at the University of Wisconsin–Madison Biotechnology and Gene Expression Center.

### Phylogenetic tree reconstruction of the Argonaute and PIWI proteins.

The sequences of annotated molluscan Argonaute-like and PIWI-like proteins were obtained from NCBI. *E. scolopes* sequences were obtained from the reference transcriptome (30) by blastx (88), and translated to amino acid sequences by ExPASy (89). Protein sequences were aligned with MAFFT (90) and trimmed for sites with over 50% gaps using trimAl (91) before tree reconstruction. A phylogenetic tree was produced with RAxML using the PROTGAMMAWAG model (92). Support values were generated by 1,000 bootstrap pseudoreplications.

To obtain light organ expression levels of miRNA machinery proteins, the 24-h light organ transcriptome was downloaded from the SRA database (accession no. PRJNA473394 [3]) and mapped against the *E. scolopes* reference transcriptome (30) with Bowtie 2 (93). Relative expression values for each tissue were estimated with RSEM software (94). A bar graph of expression values was produced with GraphPad Prism v8.00 software.

### Isolation and sequencing of RNA from light organs.

For RNA isolation, 20 juvenile light organs where pooled, and total extracted RNA was purified using the mirVana Paris kit (Invitrogen), which was followed by treatment with DNase I (Thermo Fisher Scientific). RNA concentrations were determined with a Qubit RNA BR assay kit (Invitrogen). The small RNA libraries were constructed as described above.

### Analysis of known and predicted miRNAs.

FastQC (95) was used to evaluate raw sequencing reads. The low-quality nucleotide bases and adapter contamination sequences were identified and removed with Trimmomatic (96) and Cutadapt (97). Reads ranging from 14 to 36 bp were collected for alignment with the *E. scolopes* genome (30) using miRDeep2 software (33). In addition, reads were mapped to the latest miRBase database (v22.1), allowing only one mismatch to the precursor sequence. Any miRNA already present in miRBase was designated “known,” while miRNAs uniquely identified in the squid genome were considered to be “predicted.” Only predicted miRNAs with mirDeep2 scores greater than 0 were considered further.

To identify *E. scolopes* predicted miRNAs in other mollusks, the identified precursors were mapped against the genomes of five species, *Architeuthis dux* (GenBank assembly accession no. GCA_006491835.1), Octopus bimaculoides (accession no, GCA_001194135.1), *Crassostrea gigas* (accession no. GCA_000297895.1), *Lymnaea stagnalis* (accession no. GCA_900036025.1), and *Lottia gigantea* (accession no. GCF_000327385.1), and, as outgroups, two insects, Drosophila melanogaster (accession no. GCF_000001215.4) and Bombyx mori (accession no. GCA_000151625.1).

### Differential expression analysis of miRNAs.

Identified precursors in the *E. scolopes* genome or in the miRBase database were quantified with the miRDeep2 module quantifier.pl (33). Principal-component analysis (PCA) of expression values was performed with DESeq2. The R package *edgeR* (98) was used to detect differentially expressed miRNAs among conditions. miRNAs with an adjusted *P* value of <0.05 were considered significantly differentially expressed. Heatmaps of expression values of such miRNAs, as well as a hierarchical clustering, were created with *heatmap3* (99) in the R environment.

### Target gene prediction and functional annotation.

The potential targets of the differentially expressed miRNA were obtained using miRanda (100) and the 3′ UTR regions of the reference transcriptome (30). Only targets with both a score of ≥160 and a free energy of −25 kcal/mol or less were considered. Functional annotation of the mRNA targets was performed by gene ontology (GO) mapping with Blast2GO software (101). Statistical enrichment of GO terms was determined by Fisher’s exact test, with a false-discovery rate (FDR) of <0.01 in Blast2GO, and was visualized with REVIGO (102).

### Validation of the differentially expressed miRNAs and expression analysis of their potential targets by qRT-PCR.

As described above, light organ samples from APO and SYM animals were collected 24 h after hatching and, in the case of SYM, inoculation. Total RNA was reverse transcribed with the miScript II RT kit (Qiagen) with the HiFlex buffer that allows parallel cDNA synthesis of both mRNA and miRNA. For quantification expression by qRT-PCR, a miScript SYBR green kit (Qiagen) was used in a 25-μl reaction mixture, using a LightCycler 480 (Roche) system. All reactions were performed with no-RT and no-template controls to confirm that the reaction mixtures were not contaminated. Specific primers (see Table S6 in the supplemental material) were designed with Primer3plus (103). Melting-curve analyses were performed to confirm the generation of specific PCR products. Expression analyses of miRNA candidate genes were normalized to U6 RNA expression, while mRNA candidate genes were normalized to the ribosomal protein S19. Bar graphs of expression values were produced with GraphPad Prism v8.00 software.

### Visualization by confocal microscopy of mucus secretion.

To visualize levels of mucus secretion 24 h posthatching, animals were incubated with fluorescently labeled wheat germ agglutinin (WGA; Alexa 633) (Thermo Fisher) as described in Koehler et al. (104). Actin was stained with CellTracker (Invitrogen) to visualize the tissue. Imaging was performed using a Zeiss LSM 710 upright laser-scanning confocal microscope (Carl Zeiss AG, Jena, Germany) located at the University of Hawaiʻi at Mānoa (UHM) Kewalo Marine Laboratory.

### Data availability.

The data sets generated during this study have been deposited in the NCBI SRA repository under accession numbers PRJNA629011 and PRJNA629996.
