# Supplementary material for: MicroRNA-Mediated Regulation of Initial Host Responses in a Symbiotic Organ
Source: mSystems. 2021 May 11;6(3):e00081-21. doi: 10.1128/mSystems.00081-21 (PMC8125070; doi:10.1128/mSystems.00081-21)
Supplement: TABLE S1 [file mSystems.00081-21-st001.docx]

| **Table S1.** Number of miRNA detected in squid light organ and hemolymph | | | |
| --- | --- | --- | --- |
|  | Light organ^a^ | Hemolymph^b^ | Both |
| Total: | 215 | 415 | 130 |
| - in miRBase only | 66 | 129 | 66 |
| - in *E. scolopes* genome | 149 | 286 | 64 |
| Among *E. scolopes* genome sequences: |  |  |  |
| - ‘known’ (in miRBase) | 34 | 18 | 18 |
| - ‘predicted’ (not in miRbase) | 115 | 268 | 46 |
| Among Es genome predicted sequences: |  |  |  |
| - specific to one tissue | 69 | 222 | - |
| ^a^ Light organs were dissected from *E. scolopes* (Es) juvenile animals at 24-h post hatch; the numbers of miRNA indicated are a sum of what was found in APO and SYM animals, combined.  ^b^ Hemolymph samples (two) were obtained from SYM adult squid. | | | |
