# Supplementary figures and images for: MicroRNA-Mediated Regulation of Initial Host Responses in a Symbiotic Organ
Source: mSystems. 2021 May 11;6(3):e00081-21. doi: 10.1128/mSystems.00081-21 (PMC8125070; doi:10.1128/mSystems.00081-21)

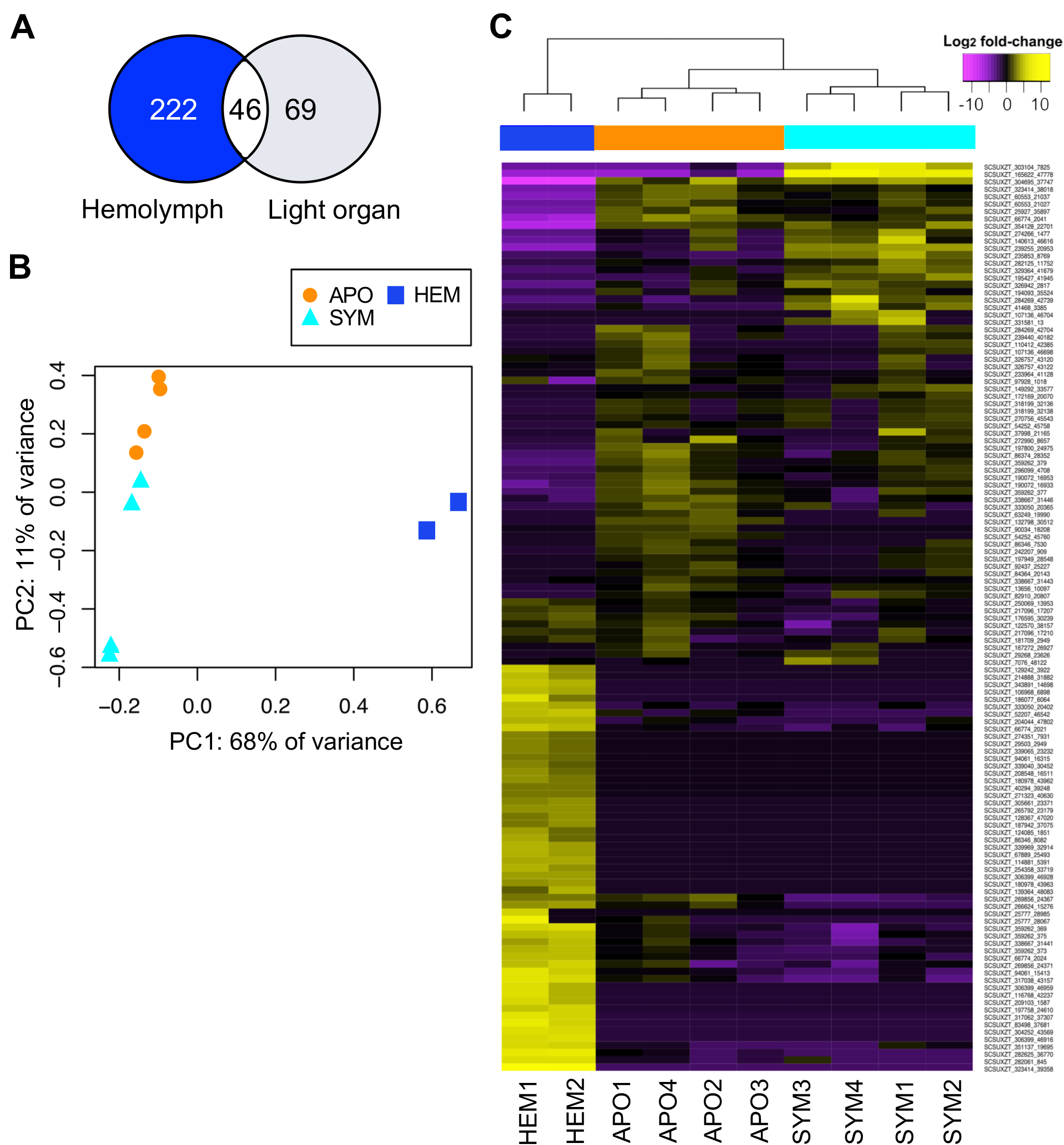

Supplement: FIG S1 [file mSystems.00081-21-sf001.tif]

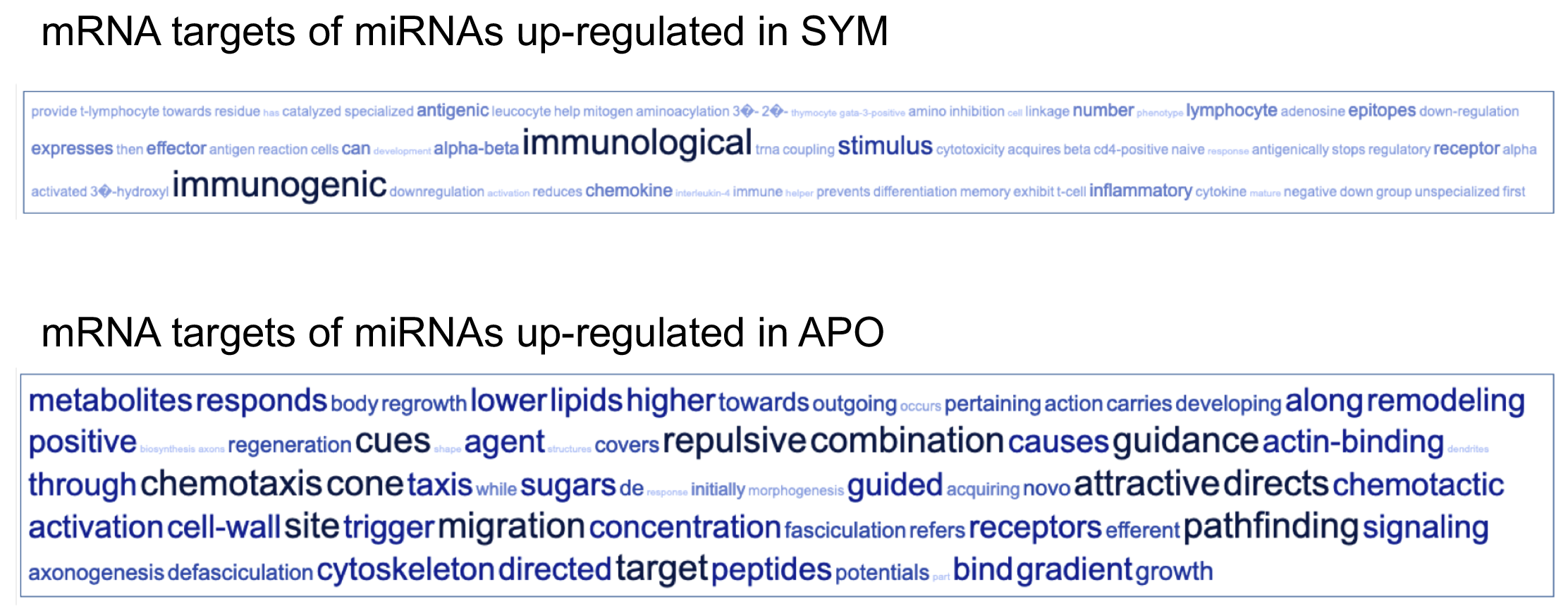

Supplement: FIG S2 [file mSystems.00081-21-sf002.tif]

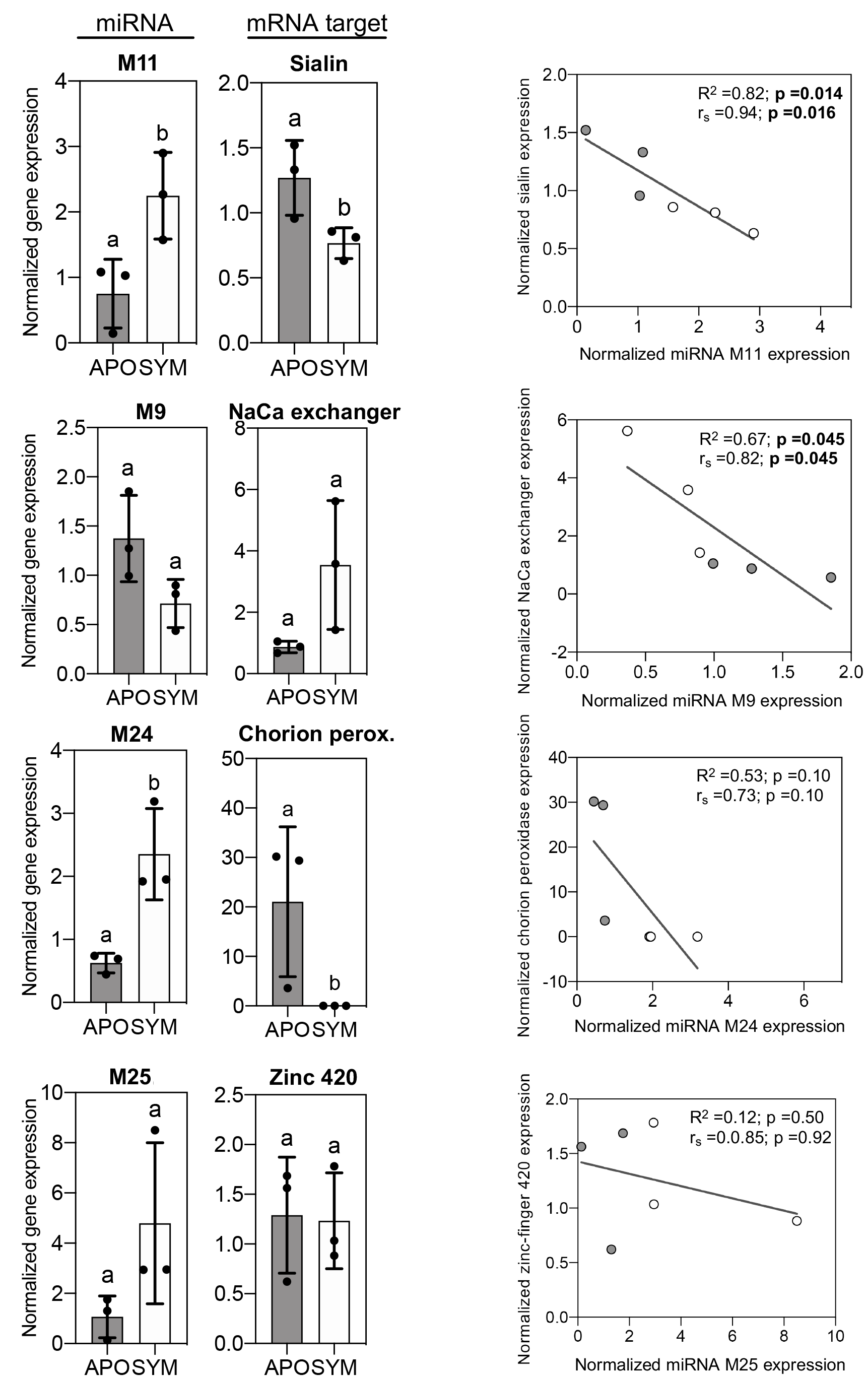

Supplement: FIG S3 [file mSystems.00081-21-sf003.tif]
