# Supplementary material for: MicroRNA-Mediated Regulation of Initial Host Responses in a Symbiotic Organ
Source: mSystems. 2021 May 11;6(3):e00081-21. doi: 10.1128/mSystems.00081-21 (PMC8125070; doi:10.1128/mSystems.00081-21)
Supplement: TABLE S2 [file mSystems.00081-21-st002.docx]

| **Table S2.** List of differentially expressed miRNA between the light organ (APO, SYM) and the hemolymph (HEM). | | | | | | |
| --- | --- | --- | --- | --- | --- | --- |
| miRNA ID | Up-regulated in | Contrast | logFC | logCPM | p Value | FDR |
| miR_323414_39358 | HEM | HEM vs SYM | 17.19 | 16.07 | 1.10E-15 | 2.27E-13 |
| miR_282625_36770 | HEM | HEM vs SYM | 14.39 | 13.46 | 1.02E-08 | 7.02E-07 |
| miR_317038_43157 | HEM | HEM vs SYM | 5.30 | 13.65 | 1.10E-06 | 4.51E-05 |
| miR_94061_15413 | HEM | HEM vs SYM | 5.20 | 13.10 | 3.57E-06 | 0.0001225 |
| miR_359262_369 | HEM | HEM vs SYM | 4.48 | 14.62 | 5.45E-06 | 0.00014065 |
| miR_83498_37681 | HEM | HEM vs SYM | 13.01 | 12.28 | 5.46E-06 | 0.00014065 |
| miR_317062_37307 | HEM | HEM vs SYM | 12.56 | 11.91 | 1.94E-05 | 0.00040027 |
| miR_306399_46916 | HEM | HEM vs SYM | 12.44 | 11.81 | 3.08E-05 | 0.00057692 |
| miR_304252_43569 | HEM | HEM vs SYM | 12.35 | 11.74 | 3.89E-05 | 0.00066854 |
| miR_52207_46542 | HEM | HEM vs SYM | 11.98 | 11.44 | 0.00014235 | 0.00225574 |
| miR_116768_42237 | HEM | HEM vs SYM | 11.72 | 11.23 | 0.00046261 | 0.00680698 |
| miR_25777_28067 | HEM | HEM vs SYM | 13.05 | 12.32 | 0.00052513 | 0.00721176 |
| miR_197758_24610 | HEM | HEM vs SYM | 11.46 | 11.02 | 0.00066473 | 0.00855843 |
| miR_306399_46959 | HEM | HEM vs SYM | 11.52 | 11.07 | 0.00077027 | 0.00933391 |
| miR_351137_19695 | HEM | HEM vs SYM | 3.31 | 12.67 | 0.00092458 | 0.01014699 |
| miR_209103_1587 | HEM | HEM vs SYM | 11.37 | 10.95 | 0.00093589 | 0.01014699 |
| miR_186077_6064 | HEM | HEM vs SYM | 11.18 | 10.80 | 0.00269222 | 0.02772983 |
| miR_269856_24371 | HEM | HEM vs SYM | 3.04 | 13.27 | 0.0032712 | 0.03149211 |
| miR_338667_31441 | HEM | HEM vs SYM | 2.70 | 15.63 | 0.00336324 | 0.03149211 |
| miR_359262_373 | HEM | HEM vs SYM | 2.44 | 13.71 | 0.0041628 | 0.03728419 |
| miR_266624_15276 | HEM | HEM vs SYM | 10.67 | 10.39 | 0.00443005 | 0.03802458 |
| miR_66774_2024 | HEM | HEM vs SYM | 3.03 | 11.36 | 0.00558386 | 0.04448055 |
| miR_106968_6898 | HEM | HEM vs SYM | 10.39 | 10.17 | 0.00561405 | 0.04448055 |
| miR_66774_2021 | HEM | HEM vs SYM | 3.17 | 12.23 | 0.00605243 | 0.04493942 |
| miR_282061_845 | HEM | HEM vs SYM | 4.30 | 12.95 | 0.00610827 | 0.04493942 |
| miR_343891_14698 | HEM | HEM vs SYM | 10.52 | 10.27 | 0.00651578 | 0.04628453 |
| miR_66774_2041 | SYM | HEM vs SYM | -11.89 | 16.31 | 1.33E-11 | 1.37E-09 |
| miR_190072_16953 | SYM | HEM vs SYM | - 6.73 | 13.33 | 7.22E-07 | 3.72E-05 |
| miR_190072_16933 | SYM | HEM vs SYM | - 7.42 | 12.04 | 9.57E-06 | 0.00021907 |
| miR_323414_39358 | HEM | HEM vs APO | 17.18 | 14.81 | 3.59E-27 | 7.40E-25 |
| miR_83498_37681 | HEM | HEM vs APO | 13.12 | 11.00 | 6.46E-10 | 2.22E-08 |
| miR_282625_36770 | HEM | HEM vs APO | 5.00 | 12.14 | 2.78E-09 | 8.17E-08 |
| miR_317062_37307 | HEM | HEM vs APO | 12.64 | 10.59 | 5.33E-09 | 1.37E-07 |
| miR_306399_46916 | HEM | HEM vs APO | 12.39 | 10.38 | 6.80E-09 | 1.56E-07 |
| miR_304252_43569 | HEM | HEM vs APO | 12.36 | 10.34 | 1.20E-08 | 2.48E-07 |
| miR_282061_845 | HEM | HEM vs APO | 4.75 | 11.52 | 4.30E-08 | 8.05E-07 |
| miR_351137_19695 | HEM | HEM vs APO | 4.66 | 11.25 | 6.59E-08 | 1.13E-06 |
| miR_116768_42237 | HEM | HEM vs APO | 11.88 | 9.94 | 1.10E-06 | 1.74E-05 |
| miR_197758_24610 | HEM | HEM vs APO | 11.37 | 9.51 | 1.50E-06 | 2.20E-05 |
| miR_209103_1587 | HEM | HEM vs APO | 11.48 | 9.60 | 1.68E-06 | 2.30E-05 |
| miR_306399_46959 | HEM | HEM vs APO | 11.66 | 9.75 | 2.45E-06 | 3.16E-05 |
| miR_94061_15413 | HEM | HEM vs APO | 3.49 | 12.02 | 5.25E-06 | 6.36E-05 |
| miR_317038_43157 | HEM | HEM vs APO | 2.96 | 12.58 | 1.93E-05 | 0.00021487 |
| miR_186077_6064 | HEM | HEM vs APO | 11.41 | 9.55 | 3.32E-05 | 0.00032595 |
| miR_343891_14698 | HEM | HEM vs APO | 10.55 | 8.83 | 4.44E-05 | 0.000416 |
| miR_106968_6898 | HEM | HEM vs APO | 10.43 | 8.73 | 7.85E-05 | 0.00070345 |
| miR_129242_3922 | HEM | HEM vs APO | 10.59 | 8.87 | 9.29E-05 | 0.00073613 |
| miR_359262_369 | HEM | HEM vs APO | 2.34 | 13.61 | 0.0001783 | 0.00136037 |
| miR_214888_31882 | HEM | HEM vs APO | 10.09 | 8.46 | 0.00027085 | 0.00199267 |
| miR_339969_32914 | HEM | HEM vs APO | 9.71 | 8.14 | 0.00032128 | 0.00208424 |
| miR_66774_2021 | HEM | HEM vs APO | 2.77 | 10.98 | 0.00046271 | 0.00287984 |
| miR_114881_5391 | HEM | HEM vs APO | 9.76 | 8.19 | 0.00048641 | 0.00287984 |
| miR_67889_25493 | HEM | HEM vs APO | 9.82 | 8.23 | 0.00049074 | 0.00287984 |
| miR_25777_28067 | HEM | HEM vs APO | 4.28 | 11.27 | 0.00050327 | 0.00287984 |
| miR_254358_33719 | HEM | HEM vs APO | 9.56 | 8.02 | 0.00081583 | 0.00430924 |
| miR_306399_46928 | HEM | HEM vs APO | 9.45 | 7.94 | 0.00084656 | 0.00435979 |
| miR_25777_28985 | HEM | HEM vs APO | 10.24 | 8.58 | 0.00169852 | 0.00833082 |
| miR_180978_43963 | HEM | HEM vs APO | 9.12 | 7.67 | 0.00239189 | 0.01145883 |
| miR_86346_8082 | HEM | HEM vs APO | 9.08 | 7.64 | 0.00305436 | 0.01429994 |
| miR_265792_23179 | HEM | HEM vs APO | 8.75 | 7.37 | 0.00372889 | 0.01685542 |
| miR_305661_23371 | HEM | HEM vs APO | 8.78 | 7.40 | 0.00376383 | 0.01685542 |
| miR_139364_48083 | HEM | HEM vs APO | 8.98 | 7.56 | 0.00453288 | 0.0195409 |
| miR_124085_1851 | HEM | HEM vs APO | 8.85 | 7.45 | 0.00741967 | 0.03056903 |
| miR_187942_37075 | HEM | HEM vs APO | 8.60 | 7.25 | 0.00783706 | 0.03151769 |
| miR_128367_47020 | HEM | HEM vs APO | 8.61 | 7.26 | 0.00795592 | 0.03151769 |
| miR_40294_39248 | HEM | HEM vs APO | 8.13 | 6.88 | 0.00987023 | 0.03836354 |
| miR_271323_40630 | HEM | HEM vs APO | 8.19 | 6.92 | 0.01012067 | 0.03860847 |
| miR_339065_23232 | HEM | HEM vs APO | 8.14 | 6.88 | 0.01063974 | 0.03985066 |
| miR_180978_43962 | HEM | HEM vs APO | 8.43 | 7.12 | 0.01279542 | 0.04706888 |
| miR_66774_2041 | APO | HEM vs APO | -13.87 | 17.13 | 5.93E-25 | 6.11E-23 |
| miR_190072_16933 | APO | HEM vs APO | - 9.74 | 13.11 | 6.22E-15 | 4.23E-13 |
| miR_190072_16953 | APO | HEM vs APO | - 7.94 | 13.34 | 8.22E-15 | 4.23E-13 |
| miR_338667_31446 | APO | HEM vs APO | - 8.40 | 11.73 | 4.80E-10 | 1.98E-08 |
| miR_326757_43122 | APO | HEM vs APO | - 5.97 | 8.98 | 1.98E-05 | 0.00021487 |
| miR_338667_31443 | APO | HEM vs APO | - 4.89 | 9.49 | 2.36E-05 | 0.00024268 |
| miR_217096_17210 | APO | HEM vs APO | - 3.22 | 15.72 | 8.73E-05 | 0.00073613 |
| miR_122570_38157 | APO | HEM vs APO | - 3.13 | 12.40 | 8.98E-05 | 0.00073613 |
| miR_97928_1018 | APO | HEM vs APO | - 4.50 | 13.83 | 0.00029208 | 0.0020408 |
| miR_217096_17207 | APO | HEM vs APO | - 2.62 | 15.37 | 0.0002972 | 0.0020408 |
| miR_326757_43120 | APO | HEM vs APO | - 4.99 | 8.84 | 0.00032376 | 0.00208424 |
| miR_250069_13953 | APO | HEM vs APO | - 2.48 | 13.68 | 0.00076531 | 0.0042609 |
| miR_269856_24367 | APO | HEM vs APO | - 2.75 | 18.13 | 0.00081334 | 0.00430924 |
| miR_176595_30239 | APO | HEM vs APO | - 2.53 | 13.54 | 0.00099567 | 0.00500263 |
| miR_181709_2949 | APO | HEM vs APO | - 2.73 | 12.27 | 0.00481693 | 0.02025079 |
