# Supplementary material for: MicroRNA-Mediated Regulation of Initial Host Responses in a Symbiotic Organ
Source: mSystems. 2021 May 11;6(3):e00081-21. doi: 10.1128/mSystems.00081-21 (PMC8125070; doi:10.1128/mSystems.00081-21)
Supplement: TABLE S3 [file mSystems.00081-21-st003.docx]

| **Table S3.** List of light-organ miRNAs that are differentially expressed with symbiosis. | | | | | |
| --- | --- | --- | --- | --- | --- |
| miRNA ID | Up-regulated in | logFC | logCPM | PValue | FDR |
| miR.235 | APO | -7.0193716 | 7.2601518 | 0.00074023 | 0.00630806 |
| miR_132798_30512 | APO | -6.2465259 | 6.6966925 | 0.00248376 | 0.01872372 |
| miR.92b.3p | APO | -5.6806940 | 8.9131998 | 4.81E-08 | 2.35E-06 |
| miR.92b | APO | -4.5972495 | 11.0951376 | 6.89E-08 | 2.60E-06 |
| miR _269856_24367 | APO | -3.4308763 | 13.5683884 | 1.32E-07 | 3.71E-06 |
| miR.92c.3p | APO | -3.3359691 | 13.5387361 | 2.67E-07 | 6.54E-06 |
| miR.92 | APO | -3.0576654 | 8.0988634 | 0.0006611 | 0.00617031 |
| miR _181921_19442 | APO | -2.5332239 | 11.0722120 | 0.00045161 | 0.00491755 |
| miR.184 | APO | -2.4389344 | 11.0524473 | 0.00058138 | 0.00569751 |
| miR.184a | APO | -2.3430371 | 11.1015178 | 0.00080084 | 0.00654017 |
| miR _124085_1851 | SYM | 2.4901879 | 10.1390290 | 0.00161612 | 0.01267041 |
| miR _274266_1477 | SYM | 3.0447821 | 9.7155302 | 0.00069497 | 0.00619153 |
| miR _326942_2817 | SYM | 3.1788374 | 9.7191797 | 0.00054497 | 0.00562182 |
| miR _282125_11752 | SYM | 3.6081553 | 7.4021469 | 6.48E-05 | 0.00079435 |
| miR _239255_20953 | SYM | 3.9762557 | 12.5593508 | 1.12E-05 | 0.00018316 |
| miR _329364_41679 | SYM | 4.0036664 | 8.4684189 | 4.37E-07 | 9.51E-06 |
| miR _140613_46616 | SYM | 4.3418067 | 11.0107201 | 3.17E-05 | 0.00041474 |
| miR _195427_41945 | SYM | 4.7700356 | 7.8986674 | 1.30E-05 | 0.00019644 |
| miR _7076_48122 | SYM | 5.4512371 | 6.1034473 | 0.00341299 | 0.02477579 |
| miR _41468_3385 | SYM | 6.0633240 | 9.3894390 | 5.15E-07 | 1.01E-05 |
| miR _235853_8769 | SYM | 6.4145140 | 9.0577155 | 3.61E-13 | 2.36E-11 |
| miR _107136_46704 | SYM | 7.4074758 | 6.9527044 | 0.00043344 | 0.00491755 |
| miR _284269_42739 | SYM | 8.0954269 | 11.8684665 | 7.97E-08 | 2.60E-06 |
| miR _331581_13 | SYM | 8.4020455 | 7.9340835 | 4.15E-06 | 7.40E-05 |
| miR _303104_7825 | SYM | 11.7237658 | 14.2725488 | 1.17E-16 | 1.15E-14 |
| miR _165622_47778 | SYM | 15.2455138 | 18.2303139 | 8.45E-27 | 1.66E-24 |
