# Supplementary material for: MicroRNA-Mediated Regulation of Initial Host Responses in a Symbiotic Organ
Source: mSystems. 2021 May 11;6(3):e00081-21. doi: 10.1128/mSystems.00081-21 (PMC8125070; doi:10.1128/mSystems.00081-21)
Supplement: TABLE S4 [file mSystems.00081-21-st004.docx]

| **Table S4.** Predicted mRNA targets of miRNAs differentially up-regulated in either the aposymbiotic (APO) or symbiotic (SYM) state. | | | | |
| --- | --- | --- | --- | --- |
| miRNA | Target ID | Target description | Target of miRNA that is up-regulated in: | GO IDs |
| miRNA_132798_30512 | c106436_f1p0_1000 | ---NA--- | APO |  |
| miRNA_132798_30512 | c30352_f1p3_2764 | eukaryotic translation initiation factor 3 subunit A-like | APO | P:GO:0001732; F:GO:0003743; C:GO:0005852; C:GO:0016282; C:GO:0033290 |
| miRNA_132798_30512 | c59220_f1p1_1923 | Regulator of nonsense transcripts 3A | APO | P:GO:0000184; F:GO:0003676 |
| miRNA_132798_30512 | c77414_f2p2_1977 | SWI/SNF-related matrix-associated actin-dependent regulator of chromatin subfamily E member 1-like | APO | F:GO:0003677; C:GO:0016514; P:GO:0043044 |
| miRNA_132798_30512 | c80199_f1p3_3312 | mediator of RNA polymerase II transcription subunit 24-like isoform X2 | APO | C:GO:0016592 |
| miRNA_132798_30512 | c82647_f1p1_1948 | myotubularin-related protein 2-like | APO | F:GO:0004725; P:GO:0035335 |
| miRNA_132798_30512 | c91345_f1p2_2469 | regulator of nonsense transcripts 3A | APO | P:GO:0000184; F:GO:0003676 |
| miRNA_132798_30512 | c92635_f1p2_2401 | PREDICTED: uncharacterized protein LOC106127467 | APO |  |
| miRNA_132798_30512 | g103017.t1 | nipped-B-like protein | APO | F:GO:0003682; C:GO:0005634; P:GO:0007049; P:GO:0010468 |
| miRNA_132798_30512 | g28146.t1 | protein HID1-like | APO |  |
| miRNA_132798_30512 | g29852.t1 | Niemann-Pick C1 protein-like | APO | P:GO:0001666; C:GO:0016020; P:GO:0030301; P:GO:0035855; P:GO:0043009; P:GO:0055113 |
| miRNA_132798_30512 | g30101.t1 | golgin subfamily A member 2-like | APO | C:GO:0005794 |
| miRNA_132798_30512 | g30991.t1 | WD repeat-containing protein 47-like isoform X1 | APO |  |
| miRNA_132798_30512 | g32522.t1 | Retrovirus-related Pol polyprotein from transposon 17.6 | APO |  |
| miRNA_132798_30512 | g34370.t1 | ATPase family AAA domain-containing protein 5-like | APO | F:GO:0005524; P:GO:0032006 |
| miRNA_132798_30512 | g36605.t1 | uncharacterized protein LOC110459911 isoform X3 | APO |  |
| miRNA_132798_30512 | g37298.t1 | cilia- and flagella-associated protein 61 | APO |  |
| miRNA_132798_30512 | g55747.t1 | hemocyte protein-glutamine gamma-glutamyltransferase-like isoform X1 | APO | F:GO:0016740 |
| miRNA_132798_30512 | g68212.t1 | PREDICTED: LOW QUALITY PROTEIN: uncharacterized protein LOC101856069 | APO |  |
| miRNA_132798_30512 | g74873.t1 | hypothetical protein OCBIM_22037054mg, partial | APO | C:GO:0016020 |
| miRNA_132798_30512 | g81269.t1 | golgin subfamily A member 3-like | APO | P:GO:0007283; C:GO:0090498 |
| miRNA_132798_30512 | g92470.t1 | hypothetical protein OCBIM_22029953mg | APO | F:GO:0003676; F:GO:0003677 |
| miRNA_132798_30512 | g95115.t1 | tyrosine-protein kinase SRK2-like | APO | F:GO:0004672; P:GO:0016310 |
| miRNA_132798_30512 | g99799.t1 | LOW QUALITY PROTEIN: uncharacterized protein LOC112561606 | APO | P:GO:0007154; C:GO:0016020; C:GO:0016021 |
| miRNA_132798_30512 | isotig06990\|m.5479 | collagen alpha-1(XI) chain-like | APO | F:GO:0005201 |
| miRNA_132798_30512 | isotig08334\|m.10070 | dystrophin-like isoform X2 | APO |  |
| miRNA_132798_30512 | TR192819\|c3_g1_i1\|m.7533 | protein SDE2 homolog | APO |  |
| miRNA_132798_30512 | TR20914\|c11_g4_i1\|m.10674 | probable syndecan | APO | P:GO:0007411; C:GO:0016021 |
| miRNA_132798_30512 | TR323284\|c1_g1_i7\|m.3002 | sorbin and SH3 domain-containing protein 1-like isoform X12 | APO | P:GO:0007015 |
| miRNA_132798_30512 | TR323817\|c12_g1_i2\|m.37639 | zinc finger E-box-binding homeobox 2-like isoform X1 | APO | F:GO:0003676 |
| miRNA_132798_30512 | TR351429\|c0_g1_i1\|m.41732 | pancreatic triacylglycerol lipase-like | APO | F:GO:0052689 |
| miRNA_132798_30512 | TR355393\|c0_g2_i1\|m.18539 | regulator of nonsense transcripts 3A | APO | P:GO:0000184; F:GO:0003676 |
| miRNA_132798_30512 | TR355393\|c0_g2_i4\|m.18541 | regulator of nonsense transcripts 3A | APO | P:GO:0000184; F:GO:0003676 |
| miRNA_132798_30512 | TR374418\|c0_g5_i2\|m.5933 | titin homolog | APO |  |
| miRNA_132798_30512 | TR445695\|c0_g2_i1\|m.32544 | PREDICTED: uncharacterized protein LOC105342567 | APO | P:GO:0007154; C:GO:0016020 |
| miRNA_132798_30512 | TR554366\|c1_g1_i1\|m.29973 | Na/Ca exchanger | APO | F:GO:0005432; P:GO:0006816; P:GO:0007154; C:GO:0016021; P:GO:0035725 |
| miRNA_132798_30512 | TR704792\|c4_g1_i1\|m.17929 | transmembrane protein 131-like | APO | C:GO:0016021 |
| miRNA_132798_30512 | TR83271\|c3_g2_i1\|m.29598 | ---NA--- | APO |  |
| miRNA_181921_19442 | c13044_f1p20_2061 | UNKNOWN | APO |  |
| miRNA_181921_19442 | c34101_f1p0_3437 | inner nuclear membrane protein Man1-like | APO | C:GO:0016020 |
| miRNA_181921_19442 | c37954_f1p14_2283 | Regulator of rDNA transcription protein 15 | APO |  |
| miRNA_181921_19442 | c40779_f1p5_2361 | probable syndecan | APO | P:GO:0007411; C:GO:0016021 |
| miRNA_181921_19442 | c46298_f1p4_3292 | ---NA--- | APO |  |
| miRNA_181921_19442 | c85139_f1p3_1995 | PREDICTED: uncharacterized protein LOC106874353 isoform X5 | APO |  |
| miRNA_181921_19442 | c93450_f1p0_3465 | ecdysone-induced protein 75B, isoforms C/D-like isoform X1 | APO | F:GO:0005488; P:GO:0050794 |
| miRNA_181921_19442 | c97609_f1p1_1986 | probable syndecan | APO | P:GO:0007411; C:GO:0016021 |
| miRNA_181921_19442 | g10287.t1 | selenocysteine lyase-like | APO | F:GO:0003824; F:GO:0005488 |
| miRNA_181921_19442 | g13736.t1 | PREDICTED: uncharacterized protein LOC106511844, partial | APO |  |
| miRNA_181921_19442 | g14889.t1 | RNA-directed DNA polymerase from mobile element jockey-like | APO | F:GO:0003676; F:GO:0003964; P:GO:0006278; F:GO:0008270 |
| miRNA_181921_19442 | g1576.t1 | chitin synthase | APO | F:GO:0000166; F:GO:0003824; C:GO:0016020 |
| miRNA_181921_19442 | g15949.t1 | hypothetical protein OCBIM_22018288mg | APO |  |
| miRNA_181921_19442 | g27282.t1 | histone-lysine N-methyltransferase SETMAR-like | APO |  |
| miRNA_181921_19442 | g31995.t1 | CUGBP Elav-like family member 3-B isoform X6 | APO |  |
| miRNA_181921_19442 | g3221.t1 | eukaryotic translation initiation factor 4B-like | APO | F:GO:0003676 |
| miRNA_181921_19442 | g47883.t1 | histone-lysine N-methyltransferase SETMAR-like | APO | F:GO:0000014; P:GO:0000729; P:GO:0000737; C:GO:0000793; F:GO:0003690; F:GO:0003697; C:GO:0005730; P:GO:0008283; P:GO:0015074; P:GO:0031297; C:GO:0035861; F:GO:0042800; F:GO:0042803; F:GO:0044547; P:GO:0044774; F:GO:0046975; P:GO:0051568; P:GO:0071157; P:GO:0097676; P:GO:2000373; P:GO:2001034; P:GO:2001251 |
| miRNA_181921_19442 | g48925.t1 | small RNA 2'-O-methyltransferase-like | APO | P:GO:0001510; F:GO:0008171; F:GO:0008173 |
| miRNA_181921_19442 | g51771.t1 | PREDICTED: uncharacterized protein LOC105334976 | APO |  |
| miRNA_181921_19442 | g56987.t1 | protein OS-9-like | APO | C:GO:0016020 |
| miRNA_181921_19442 | g64402.t1 | piggyBac transposable element-derived protein 3-like | APO |  |
| miRNA_181921_19442 | g68180.t1 | PREDICTED: uncharacterized protein LOC106633231 | APO |  |
| miRNA_181921_19442 | g7460.t1 | ATP-dependent RNA helicase cgh-1-like | APO | F:GO:0003676; F:GO:0004386; F:GO:0005524 |
| miRNA_181921_19442 | g82943.t1 | E3 ubiquitin-protein ligase HECW2 isoform X1 | APO | P:GO:0006511; P:GO:0016567; F:GO:0061630 |
| miRNA_181921_19442 | g99392.t1 | 46 kDa FK506-binding nuclear protein-like isoform X1 | APO | F:GO:0016853 |
| miRNA_181921_19442 | TR192171\|c0_g1_i1\|m.11722 | uncharacterized protein LOC111107827 isoform X7 | APO |  |
| miRNA_181921_19442 | TR20914\|c11_g4_i2\|m.10677 | probable syndecan | APO | P:GO:0007411; C:GO:0016021 |
| miRNA_181921_19442 | TR20914\|c11_g4_i4\|m.10681 | probable syndecan | APO | P:GO:0007411; C:GO:0016021 |
| miRNA_181921_19442 | TR210868\|c1_g2_i1\|m.3915 | dedicator of cytokinesis protein 9-like | APO | F:GO:0005085; P:GO:0007264 |
| miRNA_181921_19442 | TR249086\|c4_g1_i1\|m.45434 | SEC14-like protein 5 isoform X1 | APO |  |
| miRNA_181921_19442 | TR340854\|c0_g1_i1\|m.41277 | protein PAT1 homolog 1-like isoform X2 | APO | P:GO:0000290; C:GO:0016021 |
| miRNA_181921_19442 | TR434563\|c1_g2_i8\|m.23405 | microtubule-associated serine/threonine-protein kinase 3-like isoform X4 | APO | F:GO:0000287; F:GO:0004674; F:GO:0005524; P:GO:0006468 |
| miRNA_181921_19442 | TR440297\|c2_g1_i3\|m.41261 | Aminopeptidase N | APO | F:GO:0004177; P:GO:0006508; F:GO:0008237; F:GO:0008270; C:GO:0016020; C:GO:0016021 |
| miRNA_181921_19442 | TR472968\|c0_g2_i1\|m.39995 | transcription factor HES-1-like isoform X2 | APO | F:GO:0003677; C:GO:0005634; P:GO:0006355; F:GO:0046983 |
| miRNA_181921_19442 | TR484494\|c0_g2_i1\|m.46739 | ---NA--- | APO |  |
| miRNA_181921_19442 | TR544754\|c5_g1_i1\|m.45564 | ---NA--- | APO |  |
| miRNA_181921_19442 | TR578211\|c4_g1_i1\|m.6875 | ubiquitin carboxyl-terminal hydrolase 15-like | APO | F:GO:0004843; P:GO:0006511; P:GO:0016579 |
| miRNA_181921_19442 | TR578211\|c4_g1_i2\|m.6876 | ubiquitin carboxyl-terminal hydrolase 15-like | APO | F:GO:0004843; P:GO:0006511; P:GO:0016579 |
| miRNA_181921_19442 | TR603747\|c5_g2_i1\|m.23391 | pleckstrin homology domain-containing family A member 8-like | APO | C:GO:0005737; P:GO:0120009; F:GO:0120013 |
| miRNA_181921_19442 | TR662291\|c1_g1_i1\|m.40860 | ATP-dependent RNA helicase HAS1-like isoform X2 | APO | F:GO:0003723; F:GO:0004386; F:GO:0005524 |
| miRNA_181921_19442 | TRINITY_DN35533_c118_g1_i1 | basic proline-rich protein-like | APO |  |
| miRNA_269856_24367 | c11581_f1p0_3510 | ---NA--- | APO |  |
| miRNA_269856_24367 | c119358_f5p2_1296 | synaptobrevin isoform X2 | APO |  |
| miRNA_269856_24367 | c128331_f1p1_1318 | synaptobrevin-like isoform X7 | APO | C:GO:0016021; P:GO:0016192; C:GO:0030054; C:GO:0030672; C:GO:0043005 |
| miRNA_269856_24367 | c18734_f2p3_2465 | kielin/chordin-like protein | APO | P:GO:0010466; F:GO:0030414 |
| miRNA_269856_24367 | c20937_f4p4_2955 | synaptobrevin-like isoform X7 | APO | C:GO:0016021; P:GO:0016192; C:GO:0030054; C:GO:0030672; C:GO:0043005 |
| miRNA_269856_24367 | c26718_f1p5_2766 | acid phosphatase type 7 | APO | C:GO:0016020; F:GO:0016787 |
| miRNA_269856_24367 | c27289_f1p4_2242 | kielin/chordin-like protein | APO | P:GO:0010466; F:GO:0030414 |
| miRNA_269856_24367 | c43035_f1p1_1991 | CWF19-like protein 2 | APO |  |
| miRNA_269856_24367 | c44237_f1p1_1848 | synaptobrevin-like isoform X7 | APO | C:GO:0016021; P:GO:0016192; C:GO:0030054; C:GO:0030672; C:GO:0043005 |
| miRNA_269856_24367 | c54254_f1p0_2262 | hypothetical protein OCBIM_22030393mg | APO |  |
| miRNA_269856_24367 | c69472_f1p2_3359 | protein Fe65 homolog isoform X4 | APO | F:GO:0001540 |
| miRNA_269856_24367 | c70307_f1p5_1993 | KH domain-containing, RNA-binding, signal transduction-associated protein 2-like isoform X2 | APO | F:GO:0003723; C:GO:0005654; F:GO:0042802; P:GO:0051259 |
| miRNA_269856_24367 | c8040_f3p12_2996 | Kielin/chordin-like protein | APO | P:GO:0010466; C:GO:0016020; C:GO:0016021; F:GO:0030414 |
| miRNA_269856_24367 | c81768_f1p0_2846 | autophagy-related protein 9A | APO | C:GO:0016020; C:GO:0031410 |
| miRNA_269856_24367 | g13562.t1 | potassium voltage-gated channel subfamily H member 8-like | APO | F:GO:0005249; C:GO:0005887; P:GO:0034765; P:GO:0042391; P:GO:0071805 |
| miRNA_269856_24367 | g25333.t1 | MAX gene-associated protein | APO | F:GO:0046983 |
| miRNA_269856_24367 | g44067.t1 | neurogenic locus notch homolog protein 1 | APO | F:GO:0005509; C:GO:0016020; C:GO:0016021 |
| miRNA_269856_24367 | g59859.t1 | Serine/threonine-protein kinase haspin | APO | F:GO:0004672; F:GO:0005524; P:GO:0006468; F:GO:0016301; P:GO:0016310 |
| miRNA_269856_24367 | g70207.t1 | myosin VIIa | APO | F:GO:0003774; F:GO:0005524; C:GO:0016021; C:GO:0016459; F:GO:0051015 |
| miRNA_269856_24367 | g85481.t1 | neurofibromin-like isoform X4 | APO |  |
| miRNA_269856_24367 | TR214861\|c4_g3_i1\|m.18200 | monocarboxylate transporter 6 | APO | C:GO:0016020 |
| miRNA_269856_24367 | TR236563\|c4_g1_i1\|m.41411 | electroneutral sodium bicarbonate exchanger 1-like | APO | P:GO:0006820; F:GO:0008509; C:GO:0016020 |
| miRNA_269856_24367 | TR364483\|c1_g1_i1\|m.27282 | brefeldin A-inhibited guanine nucleotide-exchange protein 3-like | APO | F:GO:0005086; P:GO:0032012 |
| miRNA_269856_24367 | TR431227\|c1_g3_i1\|m.30586 | MAX gene-associated protein | APO | F:GO:0046983 |
| miRNA_269856_24367 | TR475451\|c2_g12_i1\|m.874 | carbonic anhydrase 2 | APO | F:GO:0016829; C:GO:0044464; F:GO:0046872 |
| miRNA_269856_24367 | TR561918\|c3_g2_i1\|m.7063 | zinc finger BED domain-containing protein 4-like | APO |  |
| miRNA_269856_24367 | TR593181\|c4_g1_i1\|m.32221 | protein Fe65 homolog isoform X4 | APO | F:GO:0001540 |
| miRNA_269856_24367 | TR620168\|c8_g2_i3\|m.30621 | myosin VIIa | APO | F:GO:0003774; F:GO:0005524; C:GO:0016459; F:GO:0051015 |
| miRNA_269856_24367 | TR691595\|c0_g2_i1\|m.37830 | hypothetical protein OCBIM_22038603mg | APO |  |
| miRNA_107136_46704 | c11509_f4p14_3058 | PREDICTED: uncharacterized protein LOC106874007 isoform X2 | SYM |  |
| miRNA_107136_46704 | c122871_f1p0_1230 | hypothetical protein | SYM |  |
| miRNA_107136_46704 | c13349_f1p0_1391 | ---NA--- | SYM |  |
| miRNA_107136_46704 | c14610_f1p0_2537 | gastrula zinc finger protein XlCGF26.1-like isoform X1 | SYM | F:GO:0003676 |
| miRNA_107136_46704 | c165949_f1p0_1352 | N-acetylserotonin O-methyltransferase-like protein | SYM | F:GO:0008171; P:GO:0032259 |
| miRNA_107136_46704 | c169023_f1p0_1028 | ---NA--- | SYM |  |
| miRNA_107136_46704 | c21684_f1p0_2447 | ---NA--- | SYM |  |
| miRNA_107136_46704 | c27146_f1p11_2012 | Rho GTPase | SYM | F:GO:0003924; F:GO:0005525; P:GO:0007264 |
| miRNA_107136_46704 | c33160_f3p7_3236 | probable serine/threonine-protein kinase DDB_G0267686 isoform X3 | SYM |  |
| miRNA_107136_46704 | c33722_f1p0_1869 | enolase-phosphatase E1 isoform X4 | SYM | F:GO:0046872 |
| miRNA_107136_46704 | c35093_f1p0_1232 | ---NA--- | SYM |  |
| miRNA_107136_46704 | c38166_f2p7_2627 | PREDICTED: uncharacterized protein LOC106874007 isoform X2 | SYM |  |
| miRNA_107136_46704 | c38932_f1p1_2599 | caprin-1-like isoform X1 | SYM |  |
| miRNA_107136_46704 | c41394_f1p0_2372 | gastrula zinc finger protein XlCGF8.2DB-like | SYM |  |
| miRNA_107136_46704 | c42943_f1p0_2736 | ---NA--- | SYM |  |
| miRNA_107136_46704 | c44521_f1p1_3419 | exocyst complex component 6B-like isoform X2 | SYM | C:GO:0000145; P:GO:0006904 |
| miRNA_107136_46704 | c45530_f1p0_2141 | uncharacterized transporter slc-17.2-like | SYM | C:GO:0016020; C:GO:0016021; P:GO:0055085 |
| miRNA_107136_46704 | c5078_f3p4_1453 | COP9 signalosome complex subunit 6 | SYM | P:GO:0000338; C:GO:0008180 |
| miRNA_107136_46704 | c53150_f1p6_2368 | hsc70-interacting protein | SYM | F:GO:0046983; P:GO:0051085 |
| miRNA_107136_46704 | c53390_f1p0_2349 | CDGSH iron-sulfur domain-containing protein 2 homolog A-like | SYM | C:GO:0016021; C:GO:0043231; F:GO:0051537 |
| miRNA_107136_46704 | c58041_f1p2_2832 | transforming growth factor-beta-induced protein ig-h3-like | SYM | C:GO:0005615; P:GO:0007155; P:GO:0030198; C:GO:0031012; F:GO:0050839 |
| miRNA_107136_46704 | c62347_f1p1_3083 | gastrula zinc finger protein XlCGF26.1-like | SYM | F:GO:0003676 |
| miRNA_107136_46704 | c65710_f1p0_2935 | segment polarity protein dishevelled homolog DVL-3-like isoform X4 | SYM | P:GO:0016055; P:GO:0035556 |
| miRNA_107136_46704 | c65863_f1p0_2782 | nef-associated protein 1-like | SYM | F:GO:0016301; P:GO:0016310 |
| miRNA_107136_46704 | c66015_f1p0_2879 | zinc finger protein 665-like | SYM | F:GO:0005488 |
| miRNA_107136_46704 | c67807_f1p1_2530 | tyrosine-protein kinase Abl-like isoform X1 | SYM | F:GO:0004715; F:GO:0005524; P:GO:0018108 |
| miRNA_107136_46704 | c69350_f1p1_1902 | tyrosine-protein kinase Abl-like isoform X1 | SYM | F:GO:0000166; F:GO:0004713; P:GO:0006468 |
| miRNA_107136_46704 | c70107_f2p2_3685 | innexin unc-9-like isoform X2 | SYM | C:GO:0016020 |
| miRNA_107136_46704 | c71712_f1p2_3149 | autism susceptibility gene 2 protein homolog isoform X6 | SYM |  |
| miRNA_107136_46704 | c74578_f1p1_2249 | collagen alpha-1(XXIII) chain-like | SYM |  |
| miRNA_107136_46704 | c77419_f3p3_2231 | N-acetylserotonin O-methyltransferase-like protein | SYM | F:GO:0008171; P:GO:0032259 |
| miRNA_107136_46704 | c78075_f1p0_2351 | zinc finger protein 91-like | SYM | F:GO:0003676; F:GO:0003677 |
| miRNA_107136_46704 | c81152_f1p2_2996 | potassium channel subfamily T member 1-like isoform X1 | SYM | P:GO:0006813; C:GO:0016021 |
| miRNA_107136_46704 | c82503_f1p4_2275 | nuclear receptor-binding protein-like isoform X12 | SYM | F:GO:0004672; F:GO:0005524; P:GO:0006468 |
| miRNA_107136_46704 | c8981_f3p1_1992 | lysosomal acid phosphatase | SYM | C:GO:0016020; C:GO:0016021 |
| miRNA_107136_46704 | c91290_f1p0_1831 | ---NA--- | SYM |  |
| miRNA_107136_46704 | c9350_f1p0_2775 | zinc finger protein 79 isoform X1 | SYM |  |
| miRNA_107136_46704 | c93853_f1p1_2481 | gastrula zinc finger protein XlCGF26.1-like isoform X1 | SYM |  |
| miRNA_107136_46704 | c95696_f1p0_2156 | kin of IRRE-like protein 1 | SYM | F:GO:0005509; C:GO:0016020; C:GO:0016021 |
| miRNA_107136_46704 | c98873_f1p1_2009 | ubiquitin-conjugating enzyme E2 variant 2 | SYM | C:GO:0005634; P:GO:0006281; P:GO:0016567 |
| miRNA_107136_46704 | g59987.t1 | zinc finger protein 271-like | SYM | F:GO:0005488 |
| miRNA_107136_46704 | g70782.t1 | GLTSCR1-like protein | SYM |  |
| miRNA_107136_46704 | g73017.t1 | zinc finger protein 420-like | SYM | F:GO:0000976; P:GO:0002437; P:GO:0002829; F:GO:0003676; P:GO:0045629 |
| miRNA_107136_46704 | g88658.t1 | zinc finger protein OZF-like | SYM | F:GO:0003676 |
| miRNA_107136_46704 | g93262.t1 | gastrula zinc finger protein XlCGF26.1-like | SYM | F:GO:0003676 |
| miRNA_107136_46704 | isotig01321\|m.13435 | enolase-phosphatase E1-like isoform X1 | SYM | F:GO:0046872 |
| miRNA_107136_46704 | TR109121\|c2_g1_i1\|m.29138 | zinc finger protein 271-like | SYM | F:GO:0003676 |
| miRNA_107136_46704 | TR117107\|c0_g2_i1\|m.20986 | collagen alpha-1(XII) chain-like | SYM | F:GO:0005201; F:GO:0005509; F:GO:0030246; C:GO:0062023 |
| miRNA_107136_46704 | TR14558\|c0_g2_i1\|m.2425 | PREDICTED: uncharacterized protein C6orf62 homolog | SYM |  |
| miRNA_107136_46704 | TR223006\|c4_g3_i1\|m.30712 | heparan sulfate glucosamine 3-O-sulfotransferase 5 | SYM | C:GO:0016020; F:GO:0016740 |
| miRNA_107136_46704 | TR286202\|c8_g4_i1\|m.26924 | zinc finger protein 436 | SYM | F:GO:0003676; F:GO:0003677 |
| miRNA_107136_46704 | TR313753\|c0_g1_i1\|m.27452 | gastrula zinc finger protein XlCGF26.1-like isoform X1 | SYM | F:GO:0003676 |
| miRNA_107136_46704 | TR339557\|c0_g1_i2\|m.43989 | heparan-alpha-glucosaminide N-acetyltransferase-like | SYM | C:GO:0016020 |
| miRNA_107136_46704 | TR343054\|c9_g1_i4\|m.16560 | protein phosphatase 1 regulatory subunit 27 | SYM |  |
| miRNA_107136_46704 | TR344793\|c1_g3_i1\|m.38503 | cytochrome P450 3A8-like isoform X2 | SYM | F:GO:0005488; F:GO:0016491 |
| miRNA_107136_46704 | TR358694\|c1_g5_i1\|m.2883 | oocyte zinc finger protein XlCOF6-like | SYM | F:GO:0003676 |
| miRNA_107136_46704 | TR362695\|c8_g2_i2\|m.36235 | hypothetical protein BOW45_13000, partial | SYM |  |
| miRNA_107136_46704 | TR368322\|c1_g1_i1\|m.36677 | gastrula zinc finger protein XlCGF26.1-like isoform X1 | SYM | F:GO:0003676; F:GO:0003677 |
| miRNA_107136_46704 | TR372995\|c2_g1_i4\|m.10263 | retrovirus-related Pol polyprotein from transposon 17.6 isoform X1 | SYM | F:GO:0003676; P:GO:0015074 |
| miRNA_107136_46704 | TR472128\|c4_g1_i1\|m.2427 | PREDICTED: uncharacterized protein C6orf62 homolog | SYM |  |
| miRNA_107136_46704 | TR484336\|c3_g1_i1\|m.33469 | AT-rich interactive domain-containing protein 4B | SYM | F:GO:0003677; C:GO:0005634 |
| miRNA_107136_46704 | TR496671\|c3_g1_i1\|m.24165 | RNA-binding protein MEX3B-like | SYM | F:GO:0003723 |
| miRNA_107136_46704 | TR510446\|c0_g1_i1\|m.40153 | beta-1,3-galactosyltransferase 1-like | SYM | C:GO:0000139; P:GO:0006486; F:GO:0008378; C:GO:0016021 |
| miRNA_107136_46704 | TR531433\|c2_g1_i1\|m.32639 | hypothetical protein EGW08_001840 | SYM |  |
| miRNA_107136_46704 | TR549623\|c3_g1_i1\|m.27000 | gastrula zinc finger protein XlCGF8.2DB-like | SYM | F:GO:0003676 |
| miRNA_107136_46704 | TR561909\|c7_g1_i1\|m.34333 | protein Wnt-5b-like | SYM | F:GO:0005102; C:GO:0005576; P:GO:0007275; C:GO:0016021; P:GO:0016055 |
| miRNA_107136_46704 | TR570240\|c13_g1_i1\|m.10190 | PRDM9 | SYM | F:GO:0003676; P:GO:1900111 |
| miRNA_107136_46704 | TR584256\|c1_g1_i1\|m.34944 | zinc finger protein 420-like | SYM | F:GO:0000976; P:GO:0002437; P:GO:0002829; F:GO:0003676; P:GO:0045629 |
| miRNA_107136_46704 | TR61759\|c10_g2_i1\|m.31672 | coatomer subunit delta-like | SYM | C:GO:0000139; C:GO:0005829; P:GO:0006888; P:GO:0006890; P:GO:0015031; C:GO:0030126; P:GO:0051645 |
| miRNA_107136_46704 | TR642860\|c3_g5_i1\|m.7989 | protocadherin beta-15-like isoform X1 | SYM | C:GO:0016020 |
| miRNA_107136_46704 | TR64301\|c1_g2_i2\|m.22860 | hsc70-interacting protein | SYM | F:GO:0046983; P:GO:0051085 |
| miRNA_107136_46704 | TR667759\|c6_g3_i5\|m.41397 | cysteine sulfinic acid decarboxylase-like | SYM | C:GO:0016021; F:GO:0016831; P:GO:0019752; F:GO:0030170 |
| miRNA_107136_46704 | TR705650\|c0_g1_i1\|m.38827 | sushi, von Willebrand factor type A, EGF and pentraxin domain-containing protein 1 isoform X2 | SYM | F:GO:0005509; C:GO:0016020; C:GO:0016021 |
| miRNA_107136_46704 | TR706296\|c0_g1_i1\|m.26932 | fatty acyl-CoA reductase 1-like | SYM | P:GO:0006629; C:GO:0016021; P:GO:0055114; F:GO:0080019; F:GO:0102965 |
| miRNA_107136_46704 | TR712224\|c0_g1_i1\|m.40960 | radial spoke head protein 3 homolog B-like | SYM |  |
| miRNA_107136_46704 | TR716581\|c0_g1_i1\|m.24514 | gastrula zinc finger protein XlCGF26.1-like isoform X1 | SYM | F:GO:0003700; C:GO:0005654; F:GO:0043565; P:GO:0045892; P:GO:0045944 |
| miRNA_107136_46704 | TR73903\|c8_g16_i1\|m.43557 | hypothetical protein OCBIM_22022350mg | SYM |  |
| miRNA_107136_46704 | TR8884\|c7_g2_i1\|m.36124 | zinc finger protein 271-like | SYM | F:GO:0005488 |
| miRNA_124085_1851 | c11968_f2p0_2151 | splicing factor, arginine/serine-rich 15-like | SYM | F:GO:0003676; F:GO:0003723 |
| miRNA_124085_1851 | c18827_f1p1_3014 | neuroglian-like | SYM | C:GO:0016020; C:GO:0016021 |
| miRNA_124085_1851 | c19597_f1p3_2087 | RUN and FYVE domain-containing protein 2-like isoform X4 | SYM |  |
| miRNA_124085_1851 | c47995_f1p1_2544 | neuroglian-like | SYM | C:GO:0016020; C:GO:0016021 |
| miRNA_124085_1851 | c48313_f1p3_2464 | RUN and FYVE domain-containing protein 2-like isoform X2 | SYM | F:GO:0046872 |
| miRNA_124085_1851 | c59824_f1p5_3436 | synapse-associated protein 1-like isoform X2 | SYM |  |
| miRNA_124085_1851 | c66874_f1p1_2903 | splicing factor, arginine/serine-rich 15-like | SYM | F:GO:0003723 |
| miRNA_124085_1851 | c69472_f1p2_3359 | protein Fe65 homolog isoform X4 | SYM | F:GO:0001540 |
| miRNA_124085_1851 | c71443_f2p0_2070 | RUN and FYVE domain-containing protein 2-like isoform X3 | SYM |  |
| miRNA_124085_1851 | c77807_f1p1_3491 | ankyrin repeat and BTB/POZ domain-containing protein 1-like | SYM |  |
| miRNA_124085_1851 | c81108_f1p0_3257 | probable cation-transporting ATPase 13A3 isoform X2 | SYM | F:GO:0000166; C:GO:0016020; F:GO:0016787; F:GO:0043167 |
| miRNA_124085_1851 | g104855.t1 | baculoviral IAP repeat-containing protein 7-like isoform X3 | SYM |  |
| miRNA_124085_1851 | g27374.t1 | multidrug resistance-associated protein 1 isoform X1 | SYM | F:GO:0000166; P:GO:0006810; C:GO:0016021; F:GO:0016887; F:GO:0022857 |
| miRNA_124085_1851 | g44266.t1 | Transcriptional repressor CTCF | SYM | F:GO:0003676 |
| miRNA_124085_1851 | g46283.t1 | Multiple epidermal growth factor-like domains protein 6 | SYM | F:GO:0004222; P:GO:0006508; F:GO:0008270; C:GO:0016020; C:GO:0016021 |
| miRNA_124085_1851 | g5606.t1 | cilia- and flagella-associated protein 70-like | SYM |  |
| miRNA_124085_1851 | g61908.t1 | bromodomain-containing protein 3 isoform X1 | SYM |  |
| miRNA_124085_1851 | g68051.t1 | predicted protein | SYM |  |
| miRNA_124085_1851 | g77971.t1 | endoribonuclease Dicer-like | SYM | F:GO:0003723; F:GO:0004525; P:GO:0006396; P:GO:0031047; P:GO:0090502 |
| miRNA_124085_1851 | g84152.t1 | splicing factor, arginine/serine-rich 15-like | SYM | F:GO:0003723 |
| miRNA_124085_1851 | g85182.t1 | protein dopey-1-like isoform X3 | SYM | C:GO:0005829; P:GO:0006895 |
| miRNA_124085_1851 | g90737.t1 | centrosomal protein of 295 kDa-like isoform X1 | SYM | C:GO:0005813 |
| miRNA_124085_1851 | g99507.t1 | chromodomain-helicase-DNA-binding protein 4-like isoform X9 | SYM | F:GO:0005524; P:GO:0006325; P:GO:0007051; P:GO:0007098; C:GO:0016581; F:GO:0046872 |
| miRNA_124085_1851 | TR286203\|c3_g1_i7\|m.12720 | RUN and FYVE domain-containing protein 2-like isoform X2 | SYM | F:GO:0046872 |
| miRNA_124085_1851 | TR286203\|c3_g1_i8\|m.12722 | RUN and FYVE domain-containing protein 2-like isoform X1 | SYM |  |
| miRNA_124085_1851 | TR348346\|c2_g8_i1\|m.9448 | probable cation-transporting ATPase 13A3 isoform X2 | SYM | F:GO:0000166; C:GO:0016020; F:GO:0016787; F:GO:0043167 |
| miRNA_124085_1851 | TR348346\|c2_g8_i2\|m.9450 | probable cation-transporting ATPase 13A3 isoform X2 | SYM | F:GO:0005488 |
| miRNA_124085_1851 | TR355088\|c5_g1_i1\|m.14995 | Multiple epidermal growth factor-like domains protein 6 | SYM | F:GO:0004222; P:GO:0006508; F:GO:0008270; C:GO:0016020; C:GO:0016021 |
| miRNA_124085_1851 | TR470762\|c1_g1_i1\|m.28218 | BUD13 homolog | SYM | P:GO:0000398; C:GO:0005684; C:GO:0070274 |
| miRNA_124085_1851 | TR499896\|c1_g1_i1\|m.29221 | PREDICTED: uncharacterized protein LOC106870495 | SYM | C:GO:0016020 |
| miRNA_124085_1851 | TR53960\|c0_g2_i1\|m.3413 | phosphatidylinositide phosphatase SAC2-like | SYM | F:GO:0042578 |
| miRNA_124085_1851 | TR593181\|c4_g1_i1\|m.32221 | protein Fe65 homolog isoform X4 | SYM | F:GO:0001540 |
| miRNA_124085_1851 | TR610792\|c7_g1_i1\|m.29257 | PREDICTED: uncharacterized protein LOC106870380 | SYM | P:GO:0035556 |
| miRNA_124085_1851 | TR635015\|c3_g1_i4\|m.21682 | protocadherin beta-15-like isoform X1 | SYM | F:GO:0005509; C:GO:0005886; P:GO:0007156; C:GO:0016021 |
| miRNA_124085_1851 | TR640699\|c0_g1_i1\|m.45844 | gastrula zinc finger protein XlCGF57.1-like | SYM | F:GO:0003676 |
| miRNA_124085_1851 | TR81786\|c6_g1_i2\|m.15132 | protein FAM184A-like | SYM |  |
| miRNA_140613_46616 | c96150_f1p2_2547 | transcription factor Sox-2-like | SYM | F:GO:0003677; C:GO:0005634; P:GO:0006355 |
| miRNA_140613_46616 | g10923.t1 | transcription factor Sox-2-like | SYM | F:GO:0003677; C:GO:0005634; P:GO:0006355 |
| miRNA_140613_46616 | g87678.t1 | ---NA--- | SYM |  |
| miRNA_140613_46616 | TR116179\|c0_g2_i1\|m.17608 | sialin-like isoform X3 | SYM | C:GO:0016020 |
| miRNA_140613_46616 | TR290108\|c1_g1_i1\|m.40462 | transcription factor Sox-2-like | SYM | F:GO:0003677; C:GO:0005634; P:GO:0006355 |
| miRNA_140613_46616 | TR322436\|c0_g1_i1\|m.30873 | N6-adenosine-methyltransferase 70 kDa subunit-like | SYM | C:GO:0005634; F:GO:0016422; P:GO:0080009 |
| miRNA_140613_46616 | TRINITY_DN35533_c116_g3_i1 | predicted protein | SYM |  |
| miRNA_165622_47778 | c20372_f4p19_1536 | APGWamide precursor | SYM | F:GO:0005179; C:GO:0005576; P:GO:0010469 |
| miRNA_165622_47778 | g48769.t1 | disintegrin and metalloproteinase domain-containing protein 12-like | SYM | F:GO:0008237; C:GO:0016020 |
| miRNA_165622_47778 | g55811.t1 | protein SMG8-like | SYM | P:GO:0000184 |
| miRNA_165622_47778 | TR172288\|c0_g1_i2\|m.44289 | ---NA--- | SYM |  |
| miRNA_165622_47778 | TR304300\|c0_g2_i1\|m.30104 | protein SMG8-like | SYM | P:GO:0000184 |
| miRNA_165622_47778 | TR519265\|c0_g1_i1\|m.30336 | disintegrin and metalloproteinase domain-containing protein 12-like | SYM | F:GO:0004222; P:GO:0006508; P:GO:0007229; F:GO:0008237; C:GO:0016020; C:GO:0016021 |
| miRNA_165622_47778 | TR609903\|c4_g2_i1\|m.39009 | spermatogenesis-associated protein 5-like | SYM | F:GO:0005524 |
| miRNA_195427_41945 | g3220.t1 | probable E3 ubiquitin-protein ligase MID2 isoform X3 | SYM | F:GO:0046872 |
| miRNA_195427_41945 | TR308402\|c10_g1_i1\|m.36935 | succinate dehydrogenase assembly factor 3, mitochondrial-like | SYM | C:GO:0005739; P:GO:0034553 |
| miRNA_239255_20953 | c42964_f1p16_3645 | reticulon-1-A-like isoform X3 | SYM | C:GO:0005789; C:GO:0016021 |
| miRNA_239255_20953 | TR101467\|c8_g2_i2\|m.24075 | tissue factor pathway inhibitor-like | SYM | F:GO:0004867; P:GO:0010951 |
| miRNA_274266_1477 | c123028_f1p1_1199 | enolase-phosphatase E1-like isoform X1 | SYM |  |
| miRNA_274266_1477 | c202608_f4p1_2709 | glutamine--tRNA ligase-like | SYM | F:GO:0004819; F:GO:0005524; C:GO:0005737; P:GO:0006425 |
| miRNA_274266_1477 | c48993_f1p2_2347 | dnaJ homolog subfamily B member 11 | SYM | F:GO:0005102; C:GO:0005615; C:GO:0005634; C:GO:0005788; P:GO:0006457; P:GO:0016556; P:GO:0032781; P:GO:0036498; P:GO:0050768; F:GO:0051082; P:GO:0051604 |
| miRNA_274266_1477 | c52660_f2p1_1870 | transcriptional coactivator YAP1-like isoform X3 | SYM |  |
| miRNA_274266_1477 | TR274590\|c1_g3_i1\|m.42738 | peptidyl-prolyl cis-trans isomerase-like 2 | SYM | P:GO:0006464; F:GO:0016853; F:GO:0140096 |
| miRNA_274266_1477 | TR335143\|c7_g1_i1\|m.14210 | glutamine--tRNA ligase-like | SYM | GO:0006425 |
| miRNA_274266_1477 | TR93795\|c7_g1_i1\|m.28363 | Protein SMG5 | SYM |  |
| miRNA_284269_42739 | c13636_f3p2_2017 | nuclear receptor-binding protein-like isoform X9 | SYM |  |
| miRNA_284269_42739 | c8594_f2p4_2263 | nuclear receptor-binding protein-like isoform X10 | SYM |  |
| miRNA_284269_42739 | g1267.t1 | Atrial natriuretic peptide receptor 1 | SYM | F:GO:0003824; P:GO:0009987 |
| miRNA_284269_42739 | TR171269\|c3_g1_i1\|m.21784 | E3 ubiquitin-protein ligase TRIM71 | SYM | F:GO:0008270; F:GO:0046872 |
| miRNA_284269_42739 | TR483191\|c6_g1_i1\|m.32303 | early endosome antigen 1-like isoform X1 | SYM | F:GO:0003676; F:GO:0046872 |
| miRNA_284269_42739 | TR721009\|c3_g6_i1\|m.23890 | PREDICTED: uncharacterized protein LOC105850876 | SYM |  |
| miRNA_303104_7825 | c10526_f1p1_2251 | PREDICTED: uncharacterized protein LOC106878068 | SYM | C:GO:0016021 |
| miRNA_303104_7825 | c150843_f1p0_1218 | ---NA--- | SYM |  |
| miRNA_303104_7825 | g104555.t1 | 1-phosphatidylinositol 4,5-bisphosphate phosphodiesterase epsilon-1-like | SYM | P:GO:0006629; F:GO:0008081; P:GO:0035556 |
| miRNA_303104_7825 | g10987.t1 | cysteine/serine-rich nuclear protein 3-like | SYM |  |
| miRNA_303104_7825 | g44323.t1 | small conductance calcium-activated potassium channel protein-like | SYM | F:GO:0005249; F:GO:0005516; C:GO:0008076; F:GO:0016286; P:GO:0071805 |
| miRNA_303104_7825 | g75187.t1 | Transposon TX1 uncharacterized 149 kDa protein | SYM | F:GO:0097159; F:GO:1901363 |
| miRNA_303104_7825 | g82155.t1 | serine/threonine-protein kinase LATS1-like | SYM | P:GO:0000278; F:GO:0004674; F:GO:0005524; P:GO:0006468; P:GO:0035329 |
| miRNA_303104_7825 | g87195.t1 | eukaryotic translation initiation factor 3 subunit A-like | SYM | P:GO:0001732; F:GO:0003743; C:GO:0005852; C:GO:0016282; C:GO:0033290 |
| miRNA_303104_7825 | g96398.t1 | E3 ubiquitin-protein ligase UHRF1-like | SYM | C:GO:0005634; F:GO:0046872 |
| miRNA_303104_7825 | TR261284\|c7_g2_i1\|m.18380 | protocadherin beta-15-like | SYM | P:GO:0007155; C:GO:0016020 |
| miRNA_303104_7825 | TR282736\|c0_g1_i1\|m.15860 | formin-like protein 2 | SYM | F:GO:0003779; P:GO:0008360; F:GO:0017048; P:GO:0030036 |
| miRNA_303104_7825 | TR313389\|c7_g1_i4\|m.29889 | protein piccolo-like isoform X1 | SYM | C:GO:0005623; P:GO:0006886; C:GO:0016020; F:GO:0017137 |
| miRNA_303104_7825 | TR488186\|c0_g1_i1\|m.46367 | calcium-independent protein kinase C isoform X1 | SYM | F:GO:0004697; F:GO:0005524; P:GO:0006468; C:GO:0016020; P:GO:0035556; F:GO:0046872 |
| miRNA_303104_7825 | TRINITY_DN35533_c117_g2_i1 | hypothetical protein C9926_02940, partial | SYM | C:GO:0016020; C:GO:0016021 |
| miRNA_326942_2817 | c19483_f3p1_2267 | hypothetical protein OCBIM_22035547mg, partial | SYM | F:GO:0003676; F:GO:0008270 |
| miRNA_326942_2817 | c27203_f1p4_2088 | ---NA--- | SYM |  |
| miRNA_326942_2817 | c68790_f2p4_2174 | hypothetical protein OCBIM_22035547mg, partial | SYM | F:GO:0003676; F:GO:0008270 |
| miRNA_326942_2817 | g38346.t1 | Transposon Ty3-I Gag-Pol polyprotein | SYM | F:GO:0003676; P:GO:0015074 |
| miRNA_326942_2817 | TR342930\|c3_g2_i1\|m.19272 | proline synthase co-transcribed bacterial homolog protein | SYM | F:GO:0030170 |
| miRNA_326942_2817 | TR342930\|c3_g2_i2\|m.19273 | pyridoxal phosphate homeostasis protein | SYM | F:GO:0030170 |
| miRNA_326942_2817 | TR479119\|c8_g3_i1\|m.6205 | serine-rich adhesin for platelets-like isoform X1 | SYM |  |
| miRNA_326942_2817 | TR479119\|c8_g3_i2\|m.23309 | mucin-5AC-like isoform X2 | SYM |  |
| miRNA_329364_41679 | c70064_f1p0_2038 | hemocyanin subunit 1 | SYM | F:GO:0016491; F:GO:0046872; P:GO:0055114 |
| miRNA_329364_41679 | c83101_f1p0_2632 | Transposable element Tcb2 transposase | SYM | F:GO:0003676; F:GO:0003677; P:GO:0006313; F:GO:0008270; P:GO:0015074; C:GO:0016020; C:GO:0016021; F:GO:0022857; P:GO:0055085 |
| miRNA_329364_41679 | g686.t1 | Collagen alpha-4(VI) chain | SYM | C:GO:0005581 |
| miRNA_331581_13 | isotig07931\|m.5857 | phenylalanine-4-hydroxylase-like isoform X1 | SYM | F:GO:0004505; F:GO:0005506; P:GO:0006559; P:GO:0055114 |
| miRNA_41468_3385 | TR14761\|c0_g1_i2\|m.7675 | Mediator of RNA polymerase II transcription subunit 1 | SYM | C:GO:0005634 |
| miRNA_41468_3385 | TR643063\|c5_g2_i2\|m.33313 | rho GTPase-activating protein 17-like | SYM | C:GO:0016021 |
| miRNA_7076_48122 | c21209_f1p0_1812 | kelch domain-containing protein 4-like | SYM |  |
| miRNA_7076_48122 | c46614_f1p5_2694 | chorion peroxidase-like | SYM | F:GO:0004601; P:GO:0006979; F:GO:0020037; P:GO:0055114; P:GO:0098869 |
| miRNA_7076_48122 | c57662_f1p6_3389 | focal adhesion kinase 1-like isoform X14 | SYM | F:GO:0004713; F:GO:0005524; C:GO:0005856; C:GO:0005925; P:GO:0007172; P:GO:0018108 |
| miRNA_7076_48122 | c86393_f1p26_4023 | Transketolase-like protein 2 | SYM | F:GO:0003824 |
| miRNA_7076_48122 | c97319_f1p1_3750 | ankyrin repeat domain-containing protein 17-like isoform X2 | SYM | F:GO:0003723 |
| miRNA_7076_48122 | g17489.t1 | ubiquitin carboxyl-terminal hydrolase 1-like | SYM | P:GO:0006511; P:GO:0016579; F:GO:0036459 |
| miRNA_7076_48122 | g36174.t1 | A disintegrin and metalloproteinase with thrombospondin motifs 7-like | SYM | F:GO:0008237 |
| miRNA_7076_48122 | g39704.t1 | Gag-Pol polyprotein | SYM | F:GO:0003676; F:GO:0004190; P:GO:0006508; P:GO:0015074; F:GO:0016787 |
| miRNA_7076_48122 | g77954.t1 | multiple epidermal growth factor-like domains protein 8 | SYM | F:GO:0005509; C:GO:0016020; C:GO:0016021 |
| miRNA_7076_48122 | TR281882\|c1_g1_i2\|m.23160 | E3 ubiquitin-protein ligase ubr3-like isoform X2 | SYM | F:GO:0008270; P:GO:0016567; F:GO:0061630; P:GO:0071596 |
| miRNA_7076_48122 | TR357153\|c5_g1_i1\|m.11687 | Multiple epidermal growth factor-like domains protein 8 | SYM | F:GO:0005509; C:GO:0016021 |
| miRNA_7076_48122 | TR4245\|c1_g1_i2\|m.1776 | ankyrin repeat domain-containing protein 17-like isoform X2 | SYM | F:GO:0003723 |
| miRNA_7076_48122 | TR705428\|c1_g3_i1\|m.5989 | focal adhesion kinase 1-like isoform X2 | SYM | F:GO:0004713; F:GO:0005524; C:GO:0005856; C:GO:0005925; P:GO:0007172; P:GO:0018108 |
