# Supplementary material for: MicroRNA-Mediated Regulation of Initial Host Responses in a Symbiotic Organ
Source: mSystems. 2021 May 11;6(3):e00081-21. doi: 10.1128/mSystems.00081-21 (PMC8125070; doi:10.1128/mSystems.00081-21)
Supplement: TABLE S5 [file mSystems.00081-21-st005.docx]

| \| **Table S5**. List of significantly enriched functions in predicted mRNA targets of regulated miRNAs. \| \| --- \| | | | | | | | | |
| --- | --- | --- | --- | --- | --- | --- | --- | --- | --- |
| Term ID | Description | Frequency | Targets of miRNA that are up-regulated in: | Plot  size | Log_10_ p-value | Unique-ness | Dispens-ability | Represent- ative |
| GO:0006935 | Chemotaxis | 0.48% | APO | 4.785 | 8.99 | 0.83 | 0 | 6935 |
| GO:0042330 | Taxis | 0.48% | APO | 4.789 | -8.75 | 0.84 | 0.854 | 6935 |
| GO:0009057 | Macromolecule catabolic process | 1.95% | APO | 5.399 | -5.48 | 0.623 | 0 | 9057 |
| GO:0000956 | Nuclear-transcribed mRNA catabolic process | 0.14% | APO | 4.244 | -6.63 | 0.605 | 0.871 | 9057 |
| GO:1901575 | Organic substance catabolic process | 4.61% | APO | 5.772 | -3.95 | 0.605 | 0.888 | 9057 |
| GO:0006402 | mRNA catabolic process | 0.23% | APO | 4.475 | -6.25 | 0.594 | 0.74 | 9057 |
| GO:0006401 | RNA catabolic process | 0.40% | APO | 4.705 | -5.62 | 0.586 | 0.835 | 9057 |
| GO:0000184 | Nuclear-transcribed mRNA catabolic process, nonsense-mediated decay | 0.03% | APO | 3.588 | -6.20 | 0.639 | 0.778 | 9057 |
| GO:0046700 | Heterocycle catabolic process | 1.04% | APO | 5.127 | -4.48 | 0.605 | 0.741 | 9057 |
| GO:0019439 | Aromatic compound catabolic process | 1.16% | APO | 5.174 | -4.45 | 0.602 | 0.75 | 9057 |
| GO:0044248 | Cellular catabolic process | 3.27% | APO | 5.622 | -4.23 | 0.593 | 0.785 | 9057 |
| GO:0034655 | Nucleobase-containing compound catabolic process | 0.76% | APO | 4.989 | -4.77 | 0.581 | 0.867 | 9057 |
| GO:0044270 | Cellular nitrogen compound catabolic process | 1.05% | APO | 5.127 | -4.51 | 0.605 | 0.741 | 9057 |
| GO:0044265 | Cellular macromolecule catabolic process | 1.27% | APO | 5.211 | -5.77 | 0.576 | 0.707 | 9057 |
| GO:0040011 | Locomotion | 1.00% | APO | 5.107 | -5.43 | 0.956 | 0 | 40011 |
| GO:0061564 | Axon development | 0.13% | APO | 4.217 | -8.53 | 0.338 | 0 | 61564 |
| GO:0048699 | generation of neurons | 0.37% | APO | 4.671 | -6.02 | 0.33 | 0.887 | 61564 |
| GO:0031175 | Neuron projection development | 0.22% | APO | 4.451 | -7.49 | 0.321 | 0.934 | 61564 |
| GO:0000904 | Cell morphogenesis involved in differentiation | 0.19% | APO | 4.382 | -7.99 | 0.362 | 0.794 | 61564 |
| GO:0022008 | Neurogenesis | 0.42% | APO | 4.735 | -5.62 | 0.337 | 0.924 | 61564 |
| GO:0007409 | Axonogenesis | 0.12% | APO | 4.179 | -8.64 | 0.323 | 0.97 | 61564 |
| GO:0007411 | Axon guidance | 0.07% | APO | 3.93 | -9.41 | 0.295 | 0.874 | 61564 |
| GO:0048667 | Cell morphogenesis involved in neuron differentiation | 0.15% | APO | 4.278 | -8.33 | 0.328 | 0.906 | 61564 |
| GO:0048666 | Neuron development | 0.27% | APO | 4.539 | -6.89 | 0.331 | 0.944 | 61564 |
| GO:0097485 | Neuron projection guidance | 0.07% | APO | 3.94 | -9.12 | 0.296 | 0.876 | 61564 |
| GO:0048858 | Cell projection morphogenesis | 0.17% | APO | 4.333 | -8.24 | 0.364 | 0.99 | 61564 |
| GO:0030182 | Neuron differentiation | 0.33% | APO | 4.632 | -6.25 | 0.328 | 0.963 | 61564 |
| GO:0048812 | Neuron projection morphogenesis | 0.16% | APO | 4.323 | -8.24 | 0.314 | 0.942 | 61564 |
| GO:0009056 | Catabolic process | 4.82% | APO | 5.791 | -3.51 | 0.944 | 0.02 | 9056 |
| GO:0010605 | negative regulation of macromolecule Metabolic process | 1.17% | APO | 5.176 | -5.00 | 0.826 | 0.134 | 10605 |
| GO:0009892 | Negative regulation of metabolic process | 1.26% | APO | 5.209 | -4.92 | 0.859 | 0.871 | 10605 |
| GO:0010629 | Negative regulation of gene expression | 0.78% | APO | 5.002 | -3.69 | 0.829 | 0.929 | 10605 |
| GO:0048519 | Negative regulation of biological process | 1.98% | APO | 5.406 | -3.88 | 0.915 | 0.316 | 48519 |
| GO:0009605 | Response to external stimulus | 1.37% | APO | 5.245 | -4.21 | 0.894 | 0.383 | 9605 |
| GO:0030030 | Cell projection organization | 0.61% | APO | 4.892 | -4.55 | 0.672 | 0.414 | 30030 |
| GO:0032989 | Cellular component morphogenesis | 0.99% | APO | 5.106 | -7.69 | 0.354 | 0.691 | 32989 |
| GO:0048468 | Cell development | 0.57% | APO | 4.866 | -7.03 | 0.363 | 0.796 | 32989 |
| GO:0000902 | Cell morphogenesis | 0.95% | APO | 5.085 | -6.80 | 0.339 | 0.886 | 32989 |
| GO:0030154 | Cell differentiation | 1.13% | APO | 5.162 | -4.55 | 0.369 | 0.856 | 32989 |
| GO:0007399 | Nervous system development | 0.58% | APO | 4.873 | -4.33 | 0.39 | 0.861 | 32989 |
| GO:0009653 | Anatomical structure morphogenesis | 1.54% | APO | 5.296 | -5.72 | 0.399 | 0.815 | 32989 |
| GO:0048731 | System development | 1.26% | APO | 5.21 | -3.95 | 0.371 | 0.838 | 32989 |
| GO:0048869 | Cellular developmental process | 1.90% | APO | 5.386 | -4.51 | 0.381 | 0.853 | 32989 |
| GO:0032990 | Cell part morphogenesis | 0.17% | APO | 4.349 | -8.07 | 0.404 | 0.749 | 32989 |
| GO:1901361 | Organic cyclic compound catabolic process | 1.16% | APO | 5.174 | -4.38 | 0.644 | 0.7 | 1901361 |
| GO:0002285 | Lymphocyte activation involved in immune response | 0.03% | SYM | 3.553 | -4.73 | 0.115 | 0 | 2285 |
| GO:0043367 | CD4-positive, alpha-beta T cell differentiation | 0.01% | SYM | 3.171 | -4.73 | 0.075 | 0.974 | 2285 |
| GO:0043370 | Regulation of CD4-positive, alpha-beta T cell differentiation | 0.01% | SYM | 2.894 | -4.73 | 0.064 | 0.97 | 2285 |
| GO:2000515 | Negative regulation of CD4-positive, alpha-beta T cell activation | 0.00% | SYM | 2.618 | -4.73 | 0.073 | 0.969 | 2285 |
| GO:2000514 | Regulation of CD4-positive, alpha-beta T cell activation | 0.01% | SYM | 2.967 | -4.73 | 0.087 | 0.95 | 2285 |
| GO:0043371 | Negative regulation of CD4-positive, alpha-beta T cell differentiation | 0.00% | SYM | 2.572 | -4.73 | 0.065 | 0.988 | 2285 |
| GO:0045629 | Negative regulation of T-helper 2 cell differentiation | 0.00% | SYM | 2.146 | -4.73 | 0.066 | 0.953 | 2285 |
| GO:0045622 | Regulation of T-helper cell differentiation | 0.01% | SYM | 2.782 | -4.73 | 0.055 | 0.963 | 2285 |
| GO:0045623 | Negative regulation of T-helper cell differentiation | 0.00% | SYM | 2.556 | -4.73 | 0.054 | 0.938 | 2285 |
| GO:0045628 | Regulation of T-helper 2 cell differentiation | 0.00% | SYM | 2.452 | -4.73 | 0.062 | 0.939 | 2285 |
| GO:0046634 | Regulation of alpha-beta T cell activation | 0.01% | SYM | 3.23 | -4.73 | 0.082 | 0.941 | 2285 |
| GO:0046631 | Alpha-beta T cell activation | 0.02% | SYM | 3.453 | -4.73 | 0.124 | 0.809 | 2285 |
| GO:0046632 | Alpha-beta T cell differentiation | 0.02% | SYM | 3.342 | -4.73 | 0.074 | 0.956 | 2285 |
| GO:0002683 | Negative regulation of immune system process | 0.08% | SYM | 4.012 | -3.74 | 0.144 | 0.824 | 2285 |
| GO:0046639 | Negative regulation of alpha-beta T cell differentiation | 0.00% | SYM | 2.629 | -4.73 | 0.066 | 0.962 | 2285 |
| GO:0046637 | Regulation of alpha-beta T cell differentiation | 0.01% | SYM | 3.068 | -4.73 | 0.062 | 0.931 | 2285 |
| GO:0002293 | Alpha-beta T cell differentiation involved in immune response | 0.01% | SYM | 3.085 | -4.73 | 0.066 | 0.999 | 2285 |
| GO:0002294 | CD4-positive, alpha-beta T cell differentiation involved in immune response | 0.01% | SYM | 3.076 | -4.73 | 0.064 | 0.996 | 2285 |
| GO:0002697 | Regulation of immune effector process | 0.06% | SYM | 3.915 | -4.73 | 0.152 | 0.808 | 2285 |
| GO:1903038 | Negative regulation of leukocyte cell-cell adhesion | 0.02% | SYM | 3.38 | -3.74 | 0.184 | 0.825 | 2285 |
| GO:0050863 | Regulation of T cell activation | 0.05% | SYM | 3.815 | -3.56 | 0.076 | 0.931 | 2285 |
| GO:0050866 | Negative regulation of cell activation | 0.03% | SYM | 3.586 | -4.25 | 0.156 | 0.781 | 2285 |
| GO:0045580 | Regulation of T cell differentiation | 0.02% | SYM | 3.453 | -3.56 | 0.063 | 0.941 | 2285 |
| GO:0002828 | Regulation of type 2 immune response | 0.01% | SYM | 2.792 | -4.73 | 0.198 | 0.957 | 2285 |
| GO:0035710 | CD4-positive, alpha-beta T cell activation | 0.01% | SYM | 3.22 | -4.73 | 0.103 | 0.819 | 2285 |
| GO:0045064 | T-helper 2 cell differentiation | 0.00% | SYM | 2.594 | -4.73 | 0.077 | 0.942 | 2285 |
| GO:0042093 | T-helper cell differentiation | 0.01% | SYM | 3.063 | -4.73 | 0.064 | 0.921 | 2285 |
| GO:0002263 | Cell activation involved in immune response | 0.04% | SYM | 3.737 | -4.25 | 0.14 | 0.831 | 2285 |
| GO:0002292 | T-cell differentiation involved in immune response | 0.01% | SYM | 3.128 | -4.73 | 0.08 | 0.958 | 2285 |
| GO:0002286 | T-cell activation involved in immune response | 0.02% | SYM | 3.276 | -4.73 | 0.106 | 0.914 | 2285 |
| GO:0002287 | Alpha-beta T cell activation involved in immune response | 0.01% | SYM | 3.086 | -4.73 | 0.09 | 0.95 | 2285 |
| GO:0002366 | Leukocyte activation involved in immune response | 0.04% | SYM | 3.732 | -4.25 | 0.11 | 0.972 | 2285 |
| GO:0061077 | Chaperone-mediated protein folding | 0.04% | SYM | 3.743 | -3.56 | 0.874 | 0.02 | 61077 |
| GO:0006425 | Glutaminyl-tRNA aminoacylation | 0.02% | SYM | 3.455 | -4.73 | 0.816 | 0.113 | 6425 |
| GO:0045596 | Negative regulation of cell differentiation | 0.13% | SYM | 4.214 | -4.37 | 0.312 | 0.126 | 45596 |
| GO:0007172 | Signal complex assembly | 0.00% | SYM | 2.548 | -4.25 | 0.655 | 0.198 | 7172 |
| GO:0051085 | Chaperone mediated protein folding requiring Cofactor | 0.01% | SYM | 3.117 | -3.74 | 0.866 | 0.551 | 51085 |
| GO:0051084 | 'de novo' posttranslational protein folding | 0.01% | SYM | 3.152 | -3.74 | 0.866 | 0.897 | 51085 |
| GO:0006458 | 'De novo' protein folding | 0.03% | SYM | 3.543 | -3.56 | 0.876 | 0.585 | 6458 |
| GO:0051241 | Negative regulation of multicellular organismal process | 0.21% | SYM | 4.432 | -3.58 | 0.36 | 0.599 | 51241 |
| GO:0042092 | Type-2 immune response | 0.01% | SYM | 2.885 | -4.73 | 0.279 | 0.634 | 42092 |
| GO:0002437 | Inflammatory response to antigenic stimulus | 0.01% | SYM | 2.978 | -4.73 | 0.274 | 0.641 | 2437 |
| GO:0051093 | Negative regulation of developmental process | 0.19% | SYM | 4.381 | -3.77 | 0.407 | 0.675 | 51093 |
| GO:0050777 | Negative regulation of immune response | 0.03% | SYM | 3.571 | -4.73 | 0.124 | 0.695 | 50777 |
| GO:1902106 | Negative regulation of leukocyte differentiation | 0.02% | SYM | 3.288 | -4.73 | 0.106 | 0.841 | 50777 |
| GO:0046636 | Negative regulation of alpha-beta T-cell activation | 0.01% | SYM | 2.763 | -4.73 | 0.075 | 0.892 | 50777 |
| GO:0051250 | Negative regulation of lymphocyte activation | 0.02% | SYM | 3.479 | -4.73 | 0.079 | 0.863 | 50777 |
| GO:0002698 | Negative regulation of immune effector process | 0.02% | SYM | 3.48 | -4.73 | 0.123 | 0.864 | 50777 |
| GO:0002695 | Negative regulation of leukocyte activation | 0.03% | SYM | 3.549 | -4.73 | 0.081 | 0.98 | 50777 |
| GO:0002829 | Negative regulation of type 2 immune response | 0.00% | SYM | 2.425 | -4.73 | 0.167 | 0.753 | 50777 |
| GO:0045581 | Negative regulation of T-cell differentiation | 0.01% | SYM | 2.931 | -4.73 | 0.069 | 0.912 | 50777 |
| GO:0050868 | Negative regulation of T-cell activation | 0.02% | SYM | 3.345 | -4.73 | 0.069 | 0.963 | 50777 |
| GO:0045620 | Negative regulation of lymphocyte differentiation | 0.01% | SYM | 3.018 | -4.73 | 0.08 | 0.924 | 50777 |
| GO:1903707 | Negative regulation of hemopoiesis | 0.02% | SYM | 3.474 | -3.95 | 0.113 | 0.863 | 50777 |
