# Supplementary material for: MicroRNA-Mediated Regulation of Initial Host Responses in a Symbiotic Organ
Source: mSystems. 2021 May 11;6(3):e00081-21. doi: 10.1128/mSystems.00081-21 (PMC8125070; doi:10.1128/mSystems.00081-21)
Supplement: TABLE S6 [file mSystems.00081-21-st006.docx]

| **Table S6.** List of oligonucleotides | | | |
| --- | --- | --- | --- |
| Primer ID | Description | Sequence 5' to 3' | Reference |
| **miRNA primers** | | | |
| M9 | miR_132798_30512 | CTCCTGTTCCTGCGTCAG | This study |
| M11 | miR_140613_46616 | ACCGCGGGTGCAGAT | This study |
| M19 | miR_326942_2817 | GCAGTGGAAGGGTTGTTATAG | This study |
| M25 | miR_107136_46704 | TGTGTGTTTGGGTGTGTT | This study |
| M24 | miR_7076_48122 | ACATCCACATGTTGTTGAC | This study |
| M23 | U6 snRNA | GACATATACTAAAATTGGATGGA | This study |
| **mRNA primers** | | | |
| Mucin5AC_F1q | Mucin 5AC | TGATGCCACTCTCACGGATA | This study |
| Mucin5AC_R1q |  | ACTGGAGACGGGTTGTTTTG | This study |
| Sialin-X3_F1q | Sialin X3 | GGTCATTTTGGCTGTGTCCT | This study |
| Sialin-X3_R1q |  | AACCAACTGCCAACCTTCAC | This study |
| Na/Ca_F1q | Na/Ca exchanger | TTCCCTCCTGCATGGATTAC | This study |
| Na/Ca_R1q |  | CCCAACCTCCCCAATAATCT | This study |
| zinc420_F1q | zinc finger protein 420-like | TCCATCTGGTGTGGATTTGA | This study |
| zinc420_R1q |  | GCAGCGTTAGCAATTTCCTC | This study |
| C-Peroxidase_F1q | Chorion peroxidase | CAAATGGGCCTCGTCAGTAT | This study |
| C-Peroxidase_R1q |  | GGTCGGCAGAAATTCATTGT | This study |
| 40S-qF3 | Ribosomal protein S19 | AAGGCTTTGTCCACCTTCCT | S. Moriano-Gutierrez et al. PNAS, 2019 |
| 40S-qR3 |  | TAAATGCTCCAACACCAGCA | S. Moriano-Gutierrez et al. PNAS, 2019 |
